# Stacking Interactions of Druglike Heterocycles with Nucleobases

**DOI:** 10.1021/acs.jcim.4c02420

**Published:** 2025-03-27

**Authors:** Audrey V. Conner, Lauren M. Kim, Patrick A. Fagan, Drew P. Harding, Steven E. Wheeler

**Affiliations:** Department of Chemistry, University of Georgia, Athens, Georgia 30602, United States

## Abstract

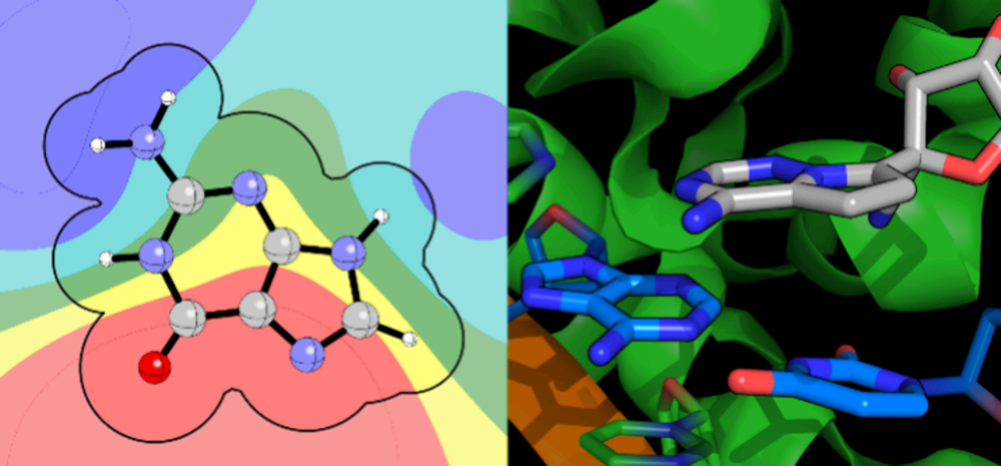

Stacking interactions contribute significantly to the interaction of small molecules with RNA, and harnessing the power of these interactions will likely prove important in the development of RNA-targeting inhibitors. To this end, we present a comprehensive computational analysis of stacking interactions between a set of 54 druglike heterocycles and the natural nucleobases. We first show that heterocycle choice can tune the strength of stacking interactions with nucleobases over a large range and that heterocycles favor stacked geometries that cluster around a discrete set of stacking loci characteristic of each nucleobase. Symmetry-adapted perturbation theory results indicate that the strengths of these interactions are modulated primarily by electrostatic and dispersion effects. Based on this, we present a multivariate predictive model of the maximum strength of stacking interactions between a given heterocycle and nucleobase that depends on molecular descriptors derived from the electrostatic potential. These descriptors can be readily computed using density functional theory or predicted directly from atom connectivity (e.g., SMILES). This model is used to predict the maximum possible stacking interactions of a set of 1854 druglike heterocycles with the natural nucleobases. Finally, we show that trivial modifications of standard (fixed-charge) molecular mechanics force fields reduce errors in predicted stacking interaction energies from around 2 kcal/mol to below 1 kcal/mol, providing a pragmatic means of predicting more reliable stacking interaction energies using existing computational workflows. We also analyze the stacking interactions between ribocil and a bacterial riboswitch, showing that two of the three aromatic heterocyclic components engage in near-optimal stacking interactions with binding site nucleobases.

## Introduction

**Figure 1:**
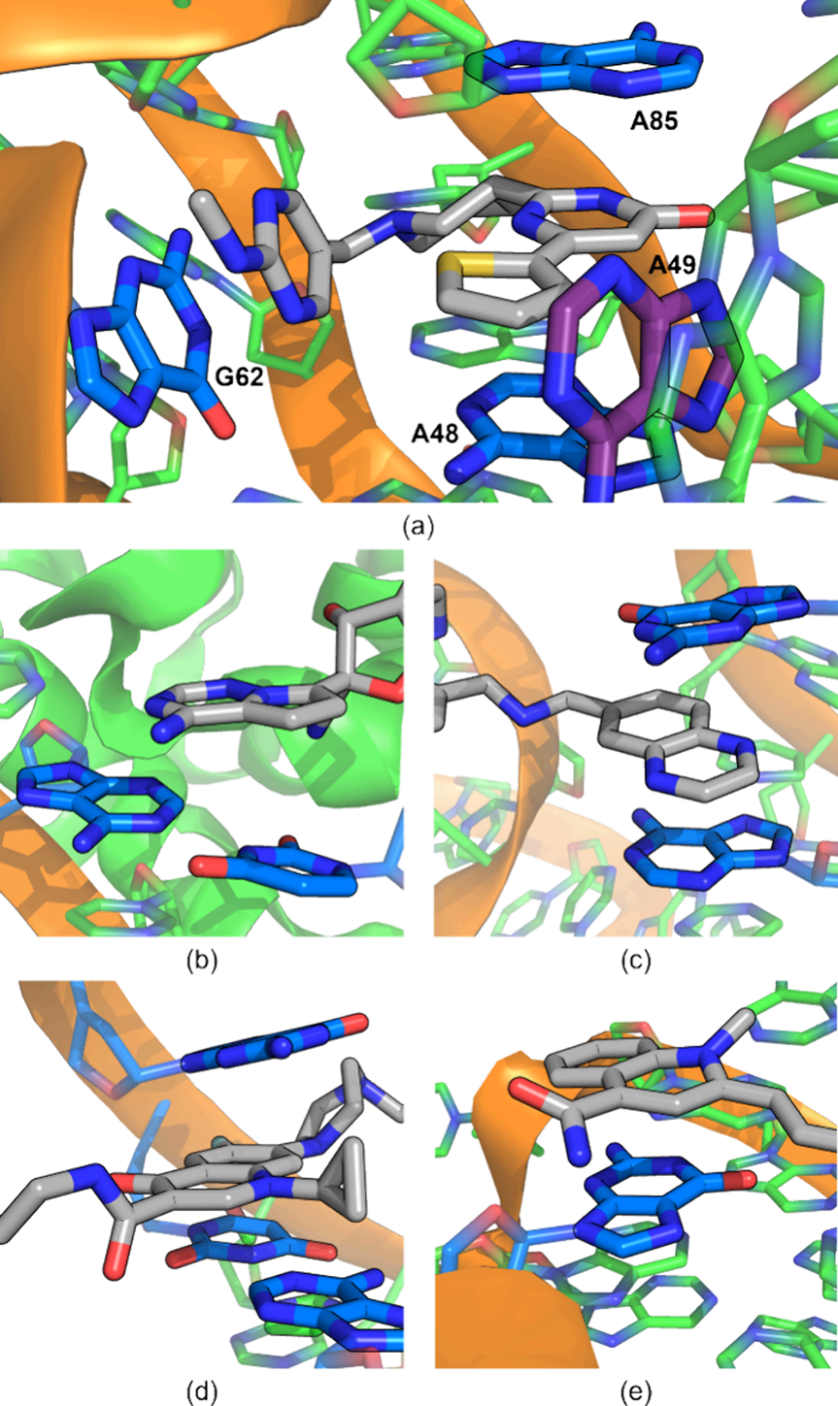
Heterocyclic components of small-molecule ligands (gray) stacking with RNA nucleobases (blue): (a) ribocil bound to the flavin mononucleotide (FMN) riboswitch of *F. nucleatum* via stacking interactions of thiophene and pyrimidone with adenines A48 and A85, respectively, and pyrimidine with guanine G62. An edge-to-face interaction between the thiophene ring and A49 (purple) also contributes to binding (PDB 5C45);^7^ (b) pyrrolotriazine fragment of the triphosphate form of remdesivir stacked with an A–U base pair in the RNA-dependent RNA polymerase from SARS-CoV2 (PDB 7BV2);^27^ (c) designed small-molecule inhibitor that binds the TPP *thiM* riboswitch in part through stacking interactions of quinoxaline between guanine and adenine (PDB 7TZU);^28^ (d) quinolinone derivative stacked between G and an A–U base pair in a model RNA hairpin with a single bulge (PDB 7FHI);^29^ (e) quinoline derivative bound to the *MYC* promoter G-quadruplex via stacking interactions with guanine (PDB 7KBW).^30^

Stacking interactions, most broadly defined as attractive noncovalent interactions between roughly parallel planar molecules,^1−3^ play vital roles in chemical and biological systems.^4−6^ The ability to predict the strength of these interactions provides a means of modulating their effect in the context of design, particularly for small-molecule pharmaceuticals. RNA lies upstream of nearly all biological functions, making the widely varying pockets within folded RNAs attractive yet mostly still elusive targets for the design of small-molecule inhibitors.^7−19^ Stacking interactions often contribute significantly to ligand binding. For instance, Figure 1 shows examples of small molecules bound to RNA in part through stacking with one or more nucleobase. The most striking of these is ribocil,^7^ which is a highly selective modulator of bacterial riboswitches. While the oxygen on the central pyrimidone ring forms two key hydrogen bonds, the binding of this inhibitor is dominated by three stacking interactions and an edge-to-face interaction (see Figure 1a). In 2020, Hargrove et al.^20^ analyzed available RNA-ligand complexes in the Protein Data Bank, finding that RNA recognition is mainly driven by stacking and hydrogen bonding interactions. More recently, Hargrove et al. showed^21^ that molecular descriptors related to stacking ability are vital in machine learning models of experimental small-molecule-RNA-binding affinities,^22^ further suggesting a determinative role of stacking interactions. Analyses of ligand–RNA interactions by Nagasawa et al.^23^ have provided further evidence of the importance of stacking interactions.

Despite the importance of stacking interactions in the design of RNA-targeting small molecules, our understanding of stacking interactions of heterocycles with nucleobases is still limited. For instance, we currently lack a means of rapidly screening potential heterocycles with respect to their ability to form highly favorable stacked dimers with nucleobases. Among other things, this hinders the development of fragment libraries for fragment-based ligand discovery (FBLD) of RNA-targeting molecules. While RNA-targeting FBLD is still in its infancy,^24,25^ recent innovations combined with advances in RNA-structure and function prediction^26^ portend imminent advances in this area. The creation of fragment libraries specifically tailored to include heterocyclic fragments with a potential to stack strongly with nucleobases should accelerate such efforts. Similarly, efforts by Hargrove et al.^96^ to devise libraries of druglike molecules tailored for RNA-binding could benefit from a means of screening heterocyclic components for their stacking abilities.

It is established that changing the number and positions of heteroatoms within a given aromatic framework can result in substantial changes in protein–ligand binding affinities.^31−37^ Bootsma et al.^38^ showed computationally that gas-phase stacking interaction energies of druglike heterocycles with the aromatic amino acid side chains Phe, Tyr, and Trp can be tuned over a large range and provided a set of guidelines for designing heterocycles with highly favorable maximum possible stacking interactions. More recently, Togo et al.^35^ studied a series of congeneric ligands for procaspase-6 that present a conserved stacking interaction involving a variable heterocyclic fragment of the ligand sandwiched between Tyr residues.^34^ This provided an experimental platform for quantifying stacking interactions of druglike heterocycles with Tyr side chains. Experimental binding free energies for this system span more than 5 kcal/mol, revealing the importance of heteroatom placement on the strength of stacking in realistic protein environments. It is expected that heterocycle choice will play a similar if not more important role in small molecules that bind RNA. However, it is not known whether the guidelines from Bootsma et al.^38^ for enhancing stacking interactions with Phe, Tyr, and Trp, or the general findings of Togo et al.,^35^ will be transferable to nucleobases.

There have been surprisingly few computational studies of stacking interactions of druglike heterocycles with the RNA or DNA nucleobases.^39−46^ While these studies have shown that reliable stacking interaction energies can be derived from DFT computations, routine exploitation of these interactions requires simpler predictive models of the strength of stacking interactions of druglike heterocycles with nucleobases coupled with a more complete understanding of these interactions. Ren and co-workers^47^ have developed a new polarizable molecular mechanics (MM) force field tailored to simulations of stacked dimers of monocyclic and bicyclic heterocycles with the five natural nucleobases and hydrogen-bonded base pairs. This force field represents a key step toward a general polarizable force field for ligand-nucleobase interactions. However, the infrastructure required for routine simulations using polarizable force fields, coupled with their increased computational cost relative to fixed-charge MM methods, limits their applicability in many contexts. Similarly, efforts to develop docking protocols for RNA-ligand binding have been plagued by the challenge of accurately capturing stacking interactions at a reasonable computational cost.^48,49^

Various approaches have been developed to predict the strength of different types of stacking interactions relevant to drug design. Initial attempts to correlate stacking interaction energies with simple molecular descriptors (e.g., molecular dipole moments) were met with limited success.^43,50−52^ In 2018, we introduced^53,54^ heterocycle descriptors based on the electrostatic potential (ESP) computed in a plane 3.25 Å from the heterocycle. Simple statistical quantities (mean, range, *etc*.) characterizing the ESP values within the projection of the van der Waals (vdW) volume of the heterocycle onto this plane provide descriptors (ESP_mean_, ESP_range_, *etc*.) that have proved useful in developing predictive models of stacking interactions.^38,54^ These descriptors follow straightforward trends based on the number and distribution of heteroatoms, allowing for the development of guidelines for modulating the strength of stacking interactions.^38^ Moreover, Bootsma and Wheeler^73^ showed that many of these descriptors can be reliably predicted directly from the connectivity of the heterocycle (e.g., SMILES), allowing rapid yet accurate predictions of potential stacking interactions of large heterocycle libraries without resorting to any quantum chemistry computations.

Harding et al.^55^ used these descriptors^54^ to develop a multivariate model that predicts the maximum possible stacking interactions of nucleobase analogs with the natural nucleobases based on ESP_range_ and the number of heavy atoms (*N*_HA_)^56^ for the nucleobase and analog. Two numerical coefficients were fit to quantum mechanical interaction energies for the 12 global minimum stacked dimers of the natural nucleobases. The resulting model predicted the strength of the maximum stacking interaction of seven nucleobase analogs with the five natural nucleobases with a root-mean-squared error (RMSE) of 0.9 kcal/mol. However, it is unclear whether this or a similar model will reliably predict stacking interactions of druglike heterocycles with the nucleobases.

Herein, we provide a comprehensive quantum chemical analysis of stacking interactions of druglike heterocycles with nucleobases. First, we show that the geometries of stacked minima for diverse heterocycles cluster around a small set of stacking loci that are characteristic of each nucleobase and that both electrostatic and dispersion interactions are vital for capturing the trend in stacking strength. Second, we show that a simple predictive model can provide robust predictions of the maximum possible strength of stacking interactions for a given heterocycle-nucleobase pair and use this model to predict the maximum stacking interaction energies of 1854 druglike heterocycles with the five natural nucleobases. Next, we demonstrate that standard MM force fields can, with trivial modifications, provide accurate interaction energies for any reasonable heterocycle-nucleobase stacking pose. This provides a pragmatic means of extracting more reliable interaction energies for different stacked poses of heterocycles with nucleobases using existing computational workflows. Finally, we analyze the stacking interactions in the binding of ribocil (Figure 1a).

## Theoretical Methods

**Figure 2:**
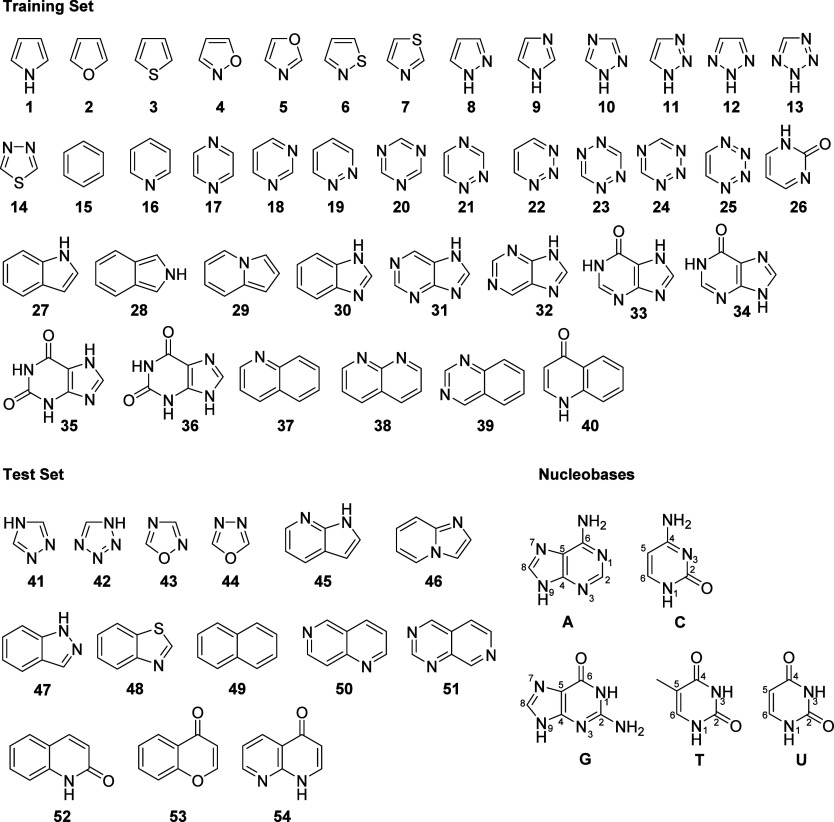
Training set and test set of druglike monocyclic and bicyclic heterocycles^38,57,58^ along with the five nucleobases (oriented with the glycosidic N in the lower left).

A set of 54 monocyclic and bicyclic heterocycles (Figure 2)^38^ was selected from common aromatic heterocycles identified by Broughton and Watson^57^ and Vitaku et al.^58^ in addition to their analogs and congeners. The set was divided into a training set (**1**–**40**) and a test set (**41**–**54**). Three nucleobase-heterocycle stacking data sets were developed based on these 54 heterocycles.

### Optimized Stacked Dimers (OSD4K)

Unique stacked energy minima (3906) for the 54 heterocycles stacked with the five nucleobases (Figure 2) consisting of all stacked local minima for each heterocycle/nucleobase pair optimized at the ωB97X-D/def2-TZVP level of theory^59,60^ under the constraint that the heavy atoms of each heterocycle remained in parallel planes.

### Nearby Random Stacked Dimers (NRSD4K)

The 3906 minima from OSD4K were displaced randomly up to ±0.5 and ±0.25 Å along the lateral and vertical axes, respectively, and rotated a random angle up to ±15° around the vertical axis.

### Fully Random Stacked Dimers (FRSD3K)

Random parallel stacked dimers (2700) had lateral displacements up to ±2.5 Å and vertical separations of 3.25 ± 0.25 Å with random orientations relative to the vertical axis.

For each of the 10,512 dimers across these three data sets, we computed binding energies, defined as the difference in energy between the optimized dimer and the optimized separated monomers, and interaction energies (energy difference between the optimized dimer and corresponding monomers in the dimer geometry) at the DLPNO-CCSD(T)/cc-pVTZ level of theory^61−65^ in ORCA 4.2.1^66^ (see the SI for details). Interaction energies were also computed at the SAPT0/jun-cc-pVDZ level of theory^67−71^ using Psi4^72^ (see the SI for details). Data for all computed dimers can be found in the SI. ESP-based descriptors for the heterocycles were taken from ref (73), while those for the nucleobases are from ref (55). Input file generation and output parsing were done using AaronTools,^74,75^ which was also used to generate the molecular structure figures in Figure 6. The ESP values plotted in Figure 6 were computed at the ωB97X-D/def2-TZVP level of theory^59,60^ using Psi4.^72^ All other DFT computations were performed using Gaussian 09.^78^

**Figure 3:**
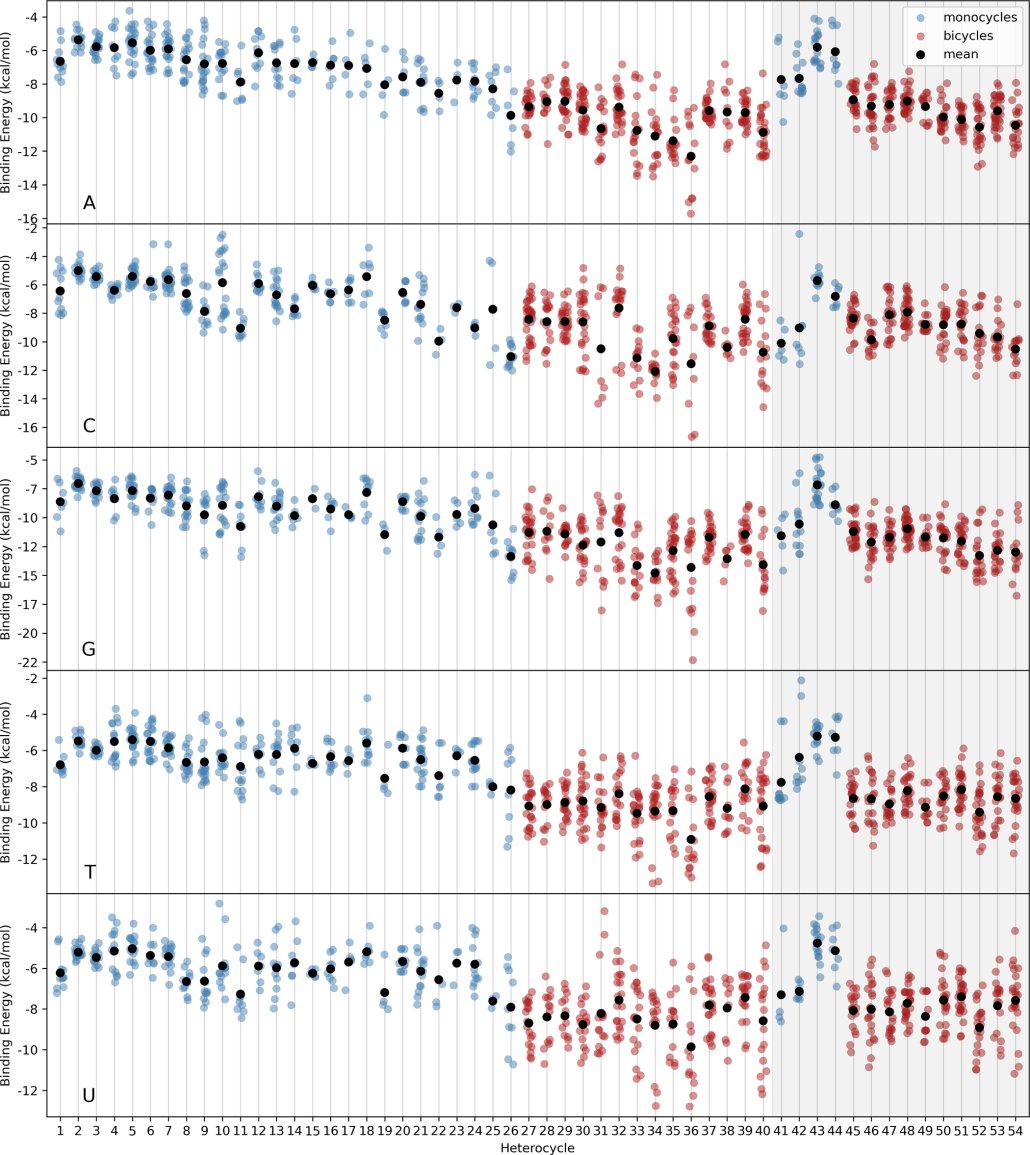
Binding energies (kcal/mol) for the 3906 stacked dimer geometries in OSD4K along with the mean value for each heterocycle/nucleobase pair. Heterocycles **1**–**40** constitute the training set, while **41**–**54** (shaded region) are the test set.

## Results and Discussion

### General Trends

**Figure 4:**
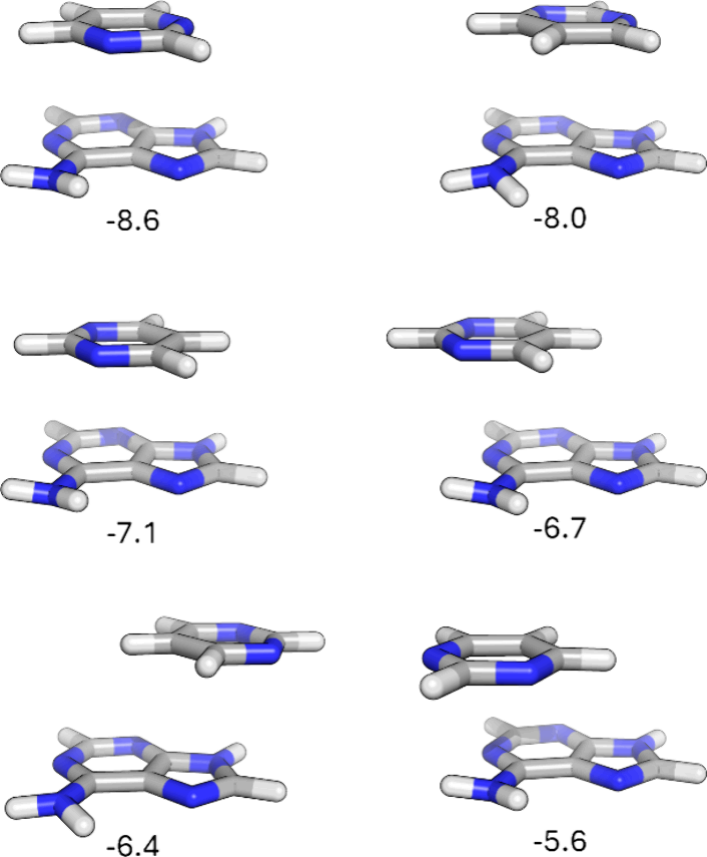
Local stacked minima of pyrimidine (**18**) with adenine; binding energies are given in kcal/mol computed at the DLPNO-CCSD(T)/cc-pvTZ level of theory.

Binding energies from OSD4K are plotted in Figure 3, separated by nucleobase and then heterocycle. The maximum binding energies for each heterocycle/nucleobase pair are listed in SI Table S1; binding energies for each heterocycle/nucleobase pair averaged over all local energy minima are listed in SI Table S2. For each heterocycle, there are anywhere from three (e.g., G···**15**) to 34 (G···**48**) local minima. For example, the six local minima for pyrimidine (**18**) stacked with adenine are shown in Figure 4, along with computed binding energies. As observed for stacking interactions with aromatic amino acid side chains,^38^ the strength of gas-phase stacking interactions with nucleobases spans a considerable range. For instance, local minima exhibit stacking interactions ranging from −2.1 kcal/mol (T···**42**) to −22.3 kcal/mol (G···**36**). For some heterocycles, the range of binding energies across local minima is very narrow, while for others, there is considerable variation based on geometry. For example, while benzene (**15**) exhibits three to six unique local stacked minima for the different nucleobases, the corresponding interaction energies span <1 kcal/mol for all but guanine. On the contrary, the interaction energies for the 18 local minima for G···**36** cover more than 12 kcal/mol. While the range of solution-phase binding enthalpies will be smaller,^79^ these data highlight the potential for modulating binding affinities over a large range through the judicious choice of heterocycle while also demonstrating the importance of heterocycle orientation in achieving maximal stacking for many heterocycles.^35^

Guanine stacks considerably more strongly than the other nucleobases, echoing previous results from Harding et al.,^55^ with the mean binding energy for the global minimum energy dimers of the 54 heterocycles with G exceeding those with A, C, T, and U by at least 3 kcal/mol (see SI Table S1). Additionally, for each heterocycle, the global minimum stacking interaction with G exceeds that of the other nucleobases. While the mean stacking interaction energies for the global minimum energy dimers are comparable for A and C (−9.9 kcal/mol for both nucleobases), the nucleobases can achieve stronger stacking as compared to T and U (−9.1 and −8.7 kcal/mol, respectively).

Overall, both the maximum and mean stacking interaction energies are correlated across the nucleobases. For example, the *r*^2^ value for the binding energies for the global minimum stacked dimers of C and G is 0.94 (unsurprisingly, *r*^2^ = 0.99 for T vs U). The least correlated are the stacking interactions with C and U (*r*^2^ = 0.81 for the maximum values, whereas for the mean values, *r*^2^ = 0.68). In other words, changes to a given heterocycle will tend to either enhance or diminish both the maximum stacking and average stacking across all five nucleobases, suggesting that achieving nucleobase selectivity through stacking alone will prove difficult without considering the orientation of the heterocycle relative to the respective nucleobases.

Turning to trends across heterocycles for a given nucleobase, we see that in general, the data for maximum stacking follow the general trends observed by Bootsma et al.^38^ For example, bicyclic heterocycles stack more strongly, on average, than monocycles (see Figure 3). However, there are many local minima for the bicyclic heterocycles whose stacking interactions are weaker than the mean value for many of the monocyclic systems. That is, while the larger heterocycles can stack more strongly than smaller ones, this requires that the bicyclic heterocycle is able to adopt an ideal stacking pose. Moreover, monocycle **26** exhibits stacking interactions that are competitive with the most strongly stacking bicyclic compounds. Similarly, maximum stacking is generally enhanced by grouping S, O, N:, and C=O groups on one side of a ring and NH groups on the other.^38^ For example, the triazines (**20**, **21**, and **22**) exhibit the expected trend in stacking strength **22** > **21** > **20** for C, G, and T (for A and U, the stacking strength is **22** ∼ **21** > **20**). Increasing the size of the heterocycle also generally leads to stronger stacking. For example, indole (**27**) and isoindole (**28**) stack 3–4 kcal/mol more strongly than pyrrole (**1**) across all nucleobases. Similar trends hold for benzimidazole (**30**) vs imidazole (**9**). The heterocycles that stack most strongly with all five nucleobases are purine derivatives **34** and **36**, as seen for stacking interactions with Phe, Tyr, and Trp side chains.^38^ It is also worth noting that for all five nucleobases, **36** stacks considerably more strongly than its tautomer **35**. Again, this matches the observations from Bootsma et al.^38^ in terms of grouping N: and C=O groups together for enhanced stacking interactions.

In total, 12 of these heterocycles exist as annular tautomeric pairs (see Table 1), providing an opportunity to assess whether stacking with a nucleobase can qualitatively change the expected tautomeric equilibrium. Previously, An et al.^43^ observed that the difference in binding energies for the two tautomers of tetrazole (**13** and **42**) stacked with A was commensurate with the tautomerization energy, suggesting that stacking could tip the scale in terms of the preferred tautomer. Table 1 shows the tautomerization energies for these six tautomer pairs for the isolated heterocycles as well as between the global minima stacked dimers with the nucleobases. In six cases, we predict a significant change in the expected tautomeric equilibrium upon stacking. For instance, while **13** is favored over its tautomer **42** by 2.0 kcal/mol in isolation, upon stacking with G, these two tautomers are essentially isoenergetic (Δ*E* = −0.1 kcal/mol). That is, even though **42** is the only tautomer expected to be present in the unbound state, when stacked with guanine, these two tautomeric states should be equally populated. Stacking with C completely swaps the identity of the preferred tautomer for this pair, with **42** now favored by 1.0 kcal/mol. Similarly, even though **33** is the favored tautomer in isolation by 0.6 kcal/mol, **34** is favored by 0.8 and 0.7 kcal/mol when stacked with T and U, respectively. Of course, these values will differ in solution, but the prospect remains that stacking with nucleobases can alter the preferred tautomeric state of some common heterocycles.

**Figure 5:**
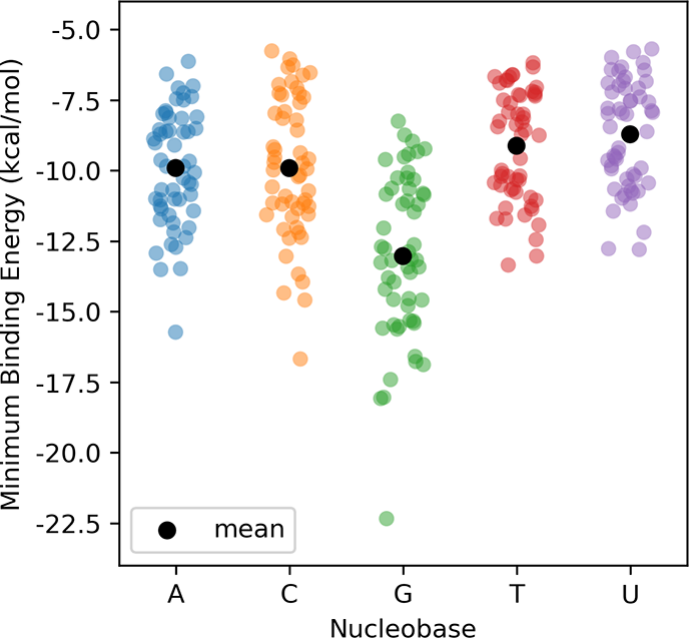
Binding energies for global minimum energy stacked dimers from OSD4K.

**Table 1:** Computed Tautomerization Energies (Δ*E*, in kcal/mol) for the Isolated Heterocycle [Δ*E*(het)] and the Heterocycle Stacked with Each of the Five Nucleobases in the Corresponding Global Minimum Energy Geometry [Δ*E*(het^···^nuc)]

		Δ*E*(het^···^nuc)				
tautomer pair	Δ*E*(het)	A	C	G	T	U
**10** → **41**	6.5	5.0	4.4	3.4	5.3	5.4
**11** → **12**	–4.4	–3.5	–1.1	–0.3	–2.9	–3.0
**13** → **42**	2.0	1.5	–1.0	–0.1	2.1	2.4
**31** → **32**	–4.0	–2.4	–1.9	–1.4	–2.8	–3.3
**33** → **34**	0.6	0.6	0.4	0.1	–0.8	–0.7
**35** → **36**	8.6	5.6	5.0	2.9	7.3	6.6

#### Geometries

**Figure 6:**
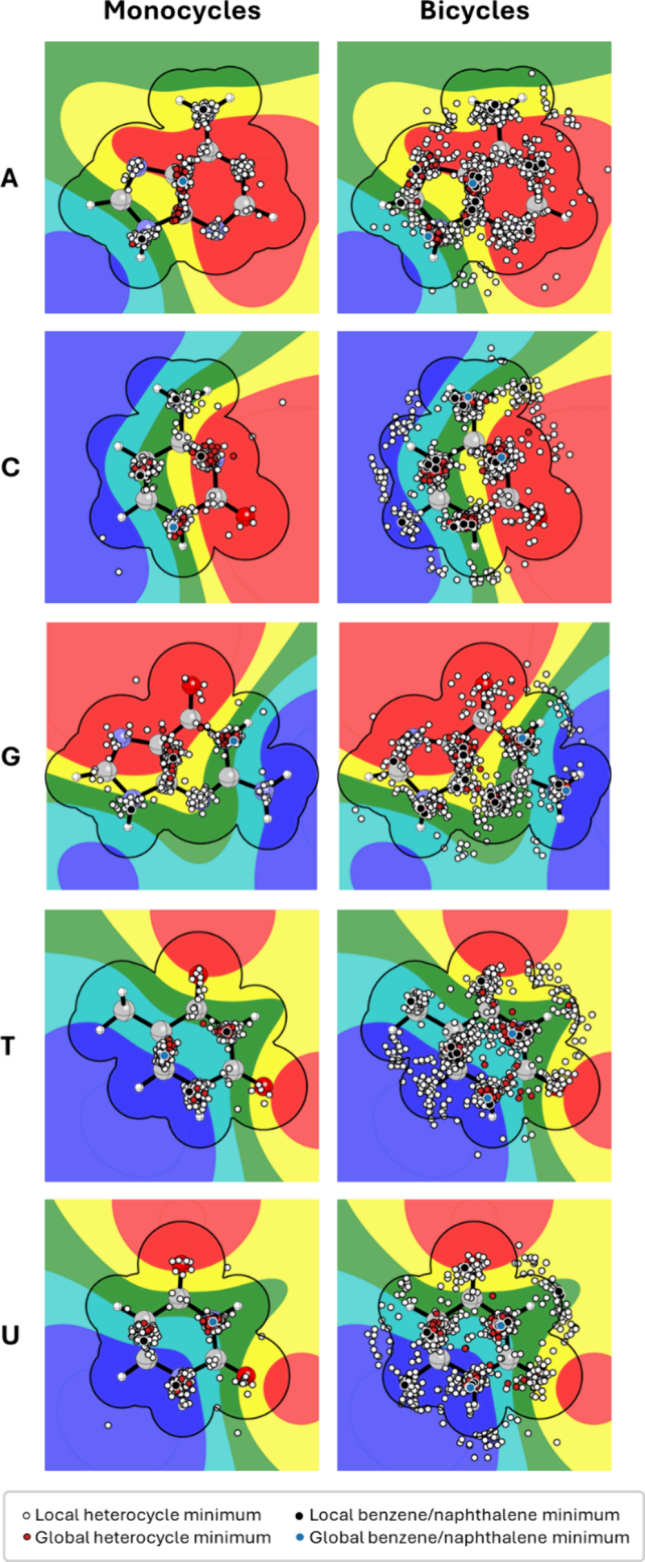
Locations of the ring centroids (circles) for optimized dimers of monocyclic (left) and bicyclic (right) heterocycles with the nucleobases for all local minima in OSD4K. The electrostatic potential in a plane 3.25 A above each nucleobase is plotted from red (−7.5 kcal/mol) to blue (+7.5 kcal/mol). The black outlines show the projection of the vdW volume of each nucleobase.

For each heterocycle/nucleobase pair, local stacked minima exhibit a wide range of geometries (e.g., see Figure 4). Figure 6 shows the locations of the ring centroids for the monocyclic and bicyclic heterocycles stacked with the nucleobases. These data are segregated into the global minimum energy dimer for each heterocycle/nucleobase pair (red) and the local minima (white). Also shown are the locations of the centroids for the local (black) and global (blue) stacked minima for benzene and naphthalene in the corresponding monocyclic/bicyclic plots. For each nucleobase, there are three or four centroid locations for the local stacked minima of benzene. Interestingly, the global minimum energy stacked dimers with benzene occur in qualitatively different positions over the face of each nucleobase. For example, whereas the global minimum features benzene over the central (C_4_–C_5_) bond of A, it is located over N_1_ for G. Similarly, while the global minimum benzene-cytosine dimer has benzene located over N_1_, for T and U, it is located over N_3_ and the C_5_–C_6_ bond, respectively. However, we note that for all but G, the benzene local minima span a very small range of binding energies, so there is little energetic distinction between the global and local minima.

The centroid locations for the monocyclic heterocycle-nucleobase geometries segregate into clear clusters. These stacking loci occur exclusively over atoms and bonds, including most *N* atom positions but also selected C—C bonds. Many, but not all, of these stacking loci are near the positions of local benzene minima. The centroid positions of the global minimum stacked geometries for the heterocycles are even more localized. For example, all but one global minimum geometry correspond to the heterocycle centroid located over the central C_4_—C_5_ bond of adenine, which is also the location of the benzene global minimum. Similarly, for guanine, all but two of the centroid locations of the global minimum stacked dimers occur at two stacking loci. It is worth noting that fewer geometries cluster around the exocyclic amino group in G than in A; most likely, this is due to a stronger preference for stacking over the purine core of G, as compared to A. The distribution of centroid locations for the global minimum energy dimers is slightly more dispersed for the pyrimidine bases but still clearly clusters around nitrogen atoms. The data for uracil and thymine prove instructive. Even though the locations of the benzene local and global minima for these two nucleobases differ, the stacking loci for these nucleobases are nearly identical. It is noteworthy that these stacking loci exhibit no clear correlation with values of the ESP for the nucleobases; the ring centroids are equally likely to cluster around areas of negative, positive, or neutral electrostatic potential. Overall, the data in Figure 6 indicate that the introduction of heteroatoms tends to provide relatively small perturbations from the preferred stacking geometry of benzene.

For the bicyclic systems, the centroid positions are somewhat more varied (and complicated by the fact that the bicyclic systems have more local minima and two ring centroids); however, the same general trends emerge. First, for naphthalene, one of the two centroids is in proximity to the global minimum for benzene for all but cytosine (again, for cytosine, the local minima for benzene are nearly isoenergetic). For all except guanine, the other ring centroid of naphthalene is located at one of the benzene local minima. Broadening the view to the naphthalene local minima, most of these have at least one ring centroid located at one of the stacking loci observed for the monocyclic systems. While there is more scatter in the centroid locations for the global minimum bicyclic heterocycle stacked dimers, the centroid locations still cluster primarily around the stacking loci identified for the monocyclic systems. In other words, the majority of stacked geometries feature the two ring centroids placed over atoms or bonds of the nucleobase; comparatively few geometries feature one ring out over the periphery of the nucleobase. Overall, these data indicate that for both mono- and bicyclic heterocycles, one can focus on stacking in one of the identified stacking loci and not be overly concerned about changes to a given heterocyclic framework causing significant disruption of the preferred stacked geometry.

### Energy Component Analysis

**Figure 7:**
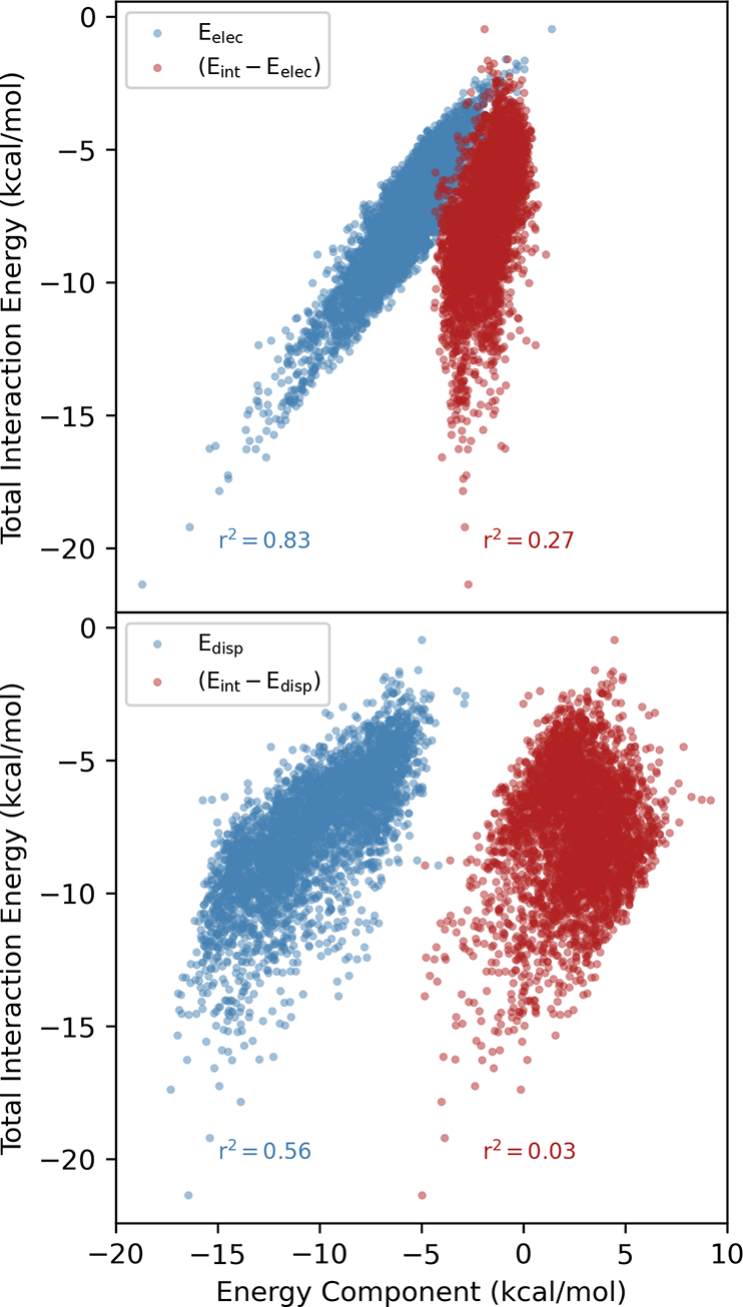
Total SAPT interaction energy vs *E*_elec_ and (*E*_int_ – *E*_elec_) (top) and *E*_disp_ and (*E*_int_ – *E*_disp_) (bottom).

SAPT computations,^67−70^ which allow for the decomposition of interaction energies into contributions from electrostatics (Elec), exchange repulsion (Exch), induction (Ind), and dispersion (Disp),^80^ were performed to gain further insight into these data. First, we looked at the correlation of each energy component with the total interaction energy each of the three data sets (see Table 2). While all four components correlate to some degree with the total interaction energy for OSD4K, the correlation with *E*_elec_ is by far the strongest (*r*^2^ = 0.83; see Table 2 and Figure 7). A simple linear regression model of the interaction energy based solely on *E*_elec_ achieves an RMSE in the total interaction energy of only 1 kcal/mol (see SI Figure S1). That all four components correlate with the total interaction energy to some degree for these optimized dimers has been observed for other noncovalent interactions^38,53,54,80^ and is primarily a reflection of the correlation of each component with the intermolecular distance (which itself correlates with the total interaction energy). To see which components are vital determinants of the total interaction energies, we looked at the correlation between the total interaction energies and the interaction energies without each component.^80^ For example, if we remove the electrostatic component from the total interaction energy (*E*_int_ – *E*_elec_), then the *r*^2^ value is only 0.27 (see Table 2) and the (*E*_int_ – *E*_elec_) values span less than 5 kcal/mol across the local minima for all 54 heterocycles and five nucleobases despite total binding energies that span a range four times that amount. This matches previous observations^38,53,54,80,81^ and reflects the balance of repulsive and attractive forces due to exchange and dispersion, respectively, near equilibrium geometries. Even though dispersion is least strongly correlated with the total interaction energies (*r*^2^ = 0.56), omitting this component of the interaction energy results in a complete loss of correlation (*r*^2^ = 0.03). However, unlike with the electrostatic component, (*E*_int_ – *E*_disp_) values cover a wide range of values, signaling that dispersion interactions alone can never capture the overall trend in stacking interactions. The behavior of the electrostatic and dispersion components can be contrasted with induction and exchange repulsion; in these cases, despite *r*^2^ values of 0.69 and 0.68, respectively, removing these components results in a stronger correlation with the total interaction energies (see Table 2 and see SI Figure S2). In summary, only electrostatic and dispersion interactions are determinative of the total interaction energies.

**Table 2:** Correlation Coefficients (*r*^2^) between Individual Components of the Interaction Energy and the Total Interaction Energy along with the Correlation Coefficients for All except the Indicated Energy Component [*r*^2^ (not)] and the Total Interaction Energy

	Elec	Exch	Ind	Disp
OSD4K				
*r*^2^	0.83	0.69	0.68	0.56
*r*^2^ (not)	0.27	0.92	0.99	0.03
NRSD4K				
*r*^2^	0.33	0.09	0.26	0.29
*r*^2^ (not)	0.04	0.35	0.93	0.02
FRSD3K				
*r*^2^	0.17	0.00	0.02	0.10
*r*^2^ (not)	0.11	0.14	0.94	0.08

The interaction energy components for NRSD4K and FRSD3K display qualitatively different behavior than seen for OSD4K, highlighting the fact that the energy-minimized stacked dimers represent special points on the respective potential energy surfaces. First, for NRSD4K, the correlations are considerably weaker than observed for the optimized dimers. For example, *E*_elec_ is still the most strongly correlated with *E*_int_, but *r*^2^ is only 0.33. That is, even modest displacements from the corresponding energy minima disrupt the balance of forces that are characteristic of the local minima, spoiling the previously strong correlations between *E*_int_ and *E*_elec_. Furthermore, while the correlation of induction with the total interaction energy is comparable to *E*_elec_ and *E*_disp_ for the data in NRSD4K, its inclusion is of no importance in capturing the total interaction energies. In the case of the fully random dimers (FRSD3K), only *E*_elec_ exhibits any correlation (*r*^2^ = 0.17) with the total interaction energy and all components except induction are vital to capture the total interaction energies.

### Predicting Optimal Stacking Interactions

Armed with knowledge that capturing electrostatics and, to a lesser extent, dispersion effects, is critical to predicting stacking strength, we developed a multivariate predictive model of the binding energies for the global minimum energy stacked dimers based on the model from Harding et al.^55^ that depends on readily computed molecular descriptors.^53,54^ The model is presented in eq 1, in which the maximum binding energy of a given heterocycle-nucleobase pair is based on the number of heavy atoms (*N*_HA_)^56^ in each system, the ESP_range_ value for the nucleobase, and the ESP_max_ value for the heterocycle.^82^ The two numerical parameters, which are not required to describe the trend in maximum interaction energies, were fit to minimize the RMSE for the training set (**1**–**40**).
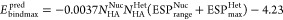
1

**Figure 8:**
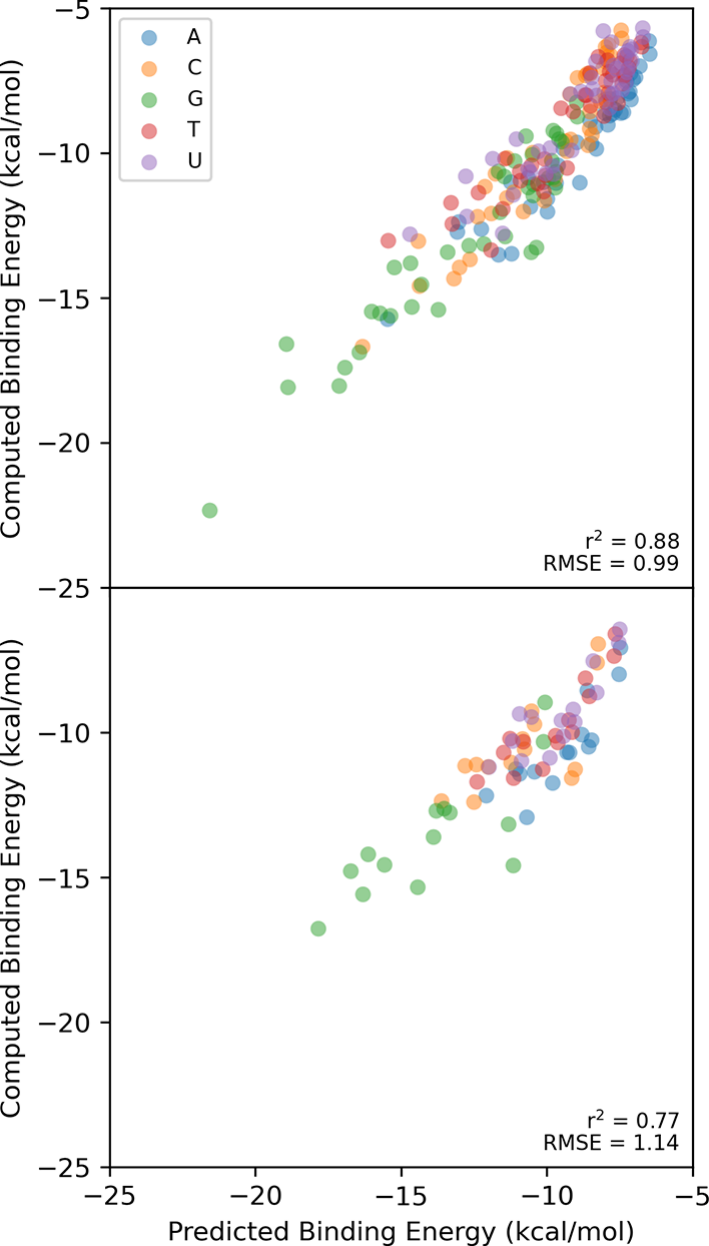
Computed binding energies for the global minimum energy dimers in OSD4K vs binding energies predicted using eq 1. (top) Training set (**1**–**40**) predicted using DFT-based descriptors; (bottom) test set (**41**–**54**) predicted using DFT-based descriptors.

The results of this fit, using DFT-computed heterocycle descriptors,^38,54^ are plotted in Figure 8 vs the computed DLPNO-CCSD(T) values. For the training set, *r*^2^ = 0.88 and RMSE = 0.99 kcal/mol; for the test set (**41**–**54**), *r*^2^ is 0.77 and RMSE 1.14 kcal/mol. Plots for each nucleobase are provided in SI Figure S3. The RMSE and *r*^2^ values are similar across all five nucleobases for the training set. The performance is slightly degraded for the test set for C and G due to the existence of a couple of outliers in both cases combined with the relatively small range of binding energies in the case of cytosine. Overall, this simple model appears to provide reliable predictions of the maximum possible stacking energy for a given heterocycle/nucleobase pair, and eq 1 can be used to rapidly screen large libraries of heterocycles with regard to their potential to stack strongly with a given nucleobase. Using SMILES-based values^73^ for ESP_max_ results in only a small increase in the RMSE and a small decrease in the correlation (see SI Figure S4), compared to the DFT-derived predictions presented in Figure 8. We note that an analogue of eq 1 based on dipole moments of the nucleobase and heterocycle,^43,50−52^ instead of ESP-derived descriptors, performs much more poorly, with *r*^2^ = 0.57 and 0.41 for the training set and test set, respectively (see SI Figure S5).

The simplicity of eq 1 enables the development of guidelines for designing heterocycles with the potential to stack strongly with the nucleobases. Bootsma et al.^38^ showed that stacking interactions with aromatic amino acid side chains are also predicted by *N*_HA_ and ESP_max_ values for the heterocycles, indicating that the general guidelines in that work will also apply to stacking with nucleobases. This was observed in the data in Figure 3 and SI Table S1. Harding et al.^55^ previously discussed the origin of the ESP_range_ values for the nucleobases in terms of the contributions from each heteroatom and functional group. This provides an explanation of the trends observed in Figure 5, namely, that G stacks more strongly than A and C stacks more strongly than either T or U, despite the similarities in size (*N*_HA_) for the purine and pyrimidine bases, respectively. In adenine, the local dipoles associated with the three imino nitrogens largely cancel, leading to a relatively small ESP_range_ value (13.1 kcal/mol). This can be contrasted with the N: adjacent to the amide carbonyl in G, whose dipoles reinforce each other and lead to a substantial ESP_range_ value (24.3 kcal/mol) and hence stronger stacking. The N: adjacent to the carbonyl in cytosine plays a similar role, leading to C having much more substantial ESP_range_ values (22.8 kcal/mol) than in either T (16.1 kcal/mol) or U (17.7 kcal/mol).^55^

**Figure 9:**
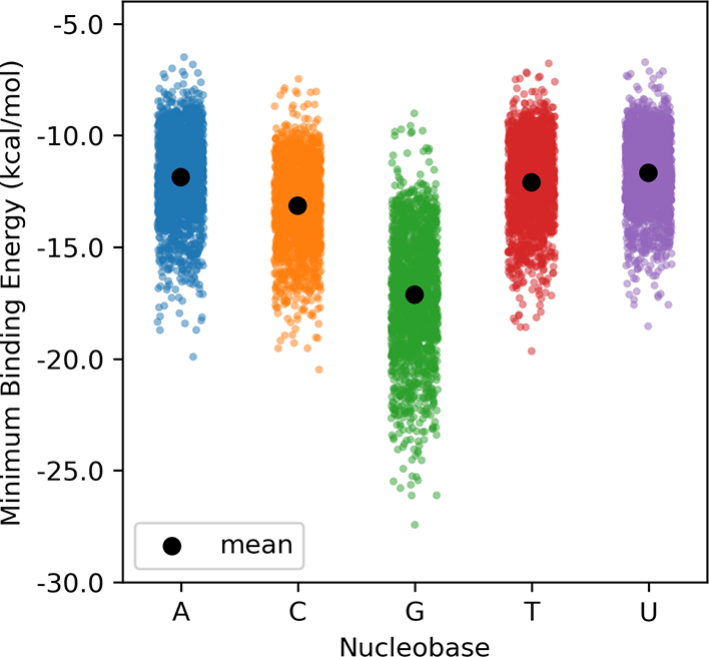
Binding energies for global minimum energy stacked dimers for a set of 1854 heterocycles (from ref (73)) with the nucleobases predicted using eq 1 with DFT-computed ESP descriptors.

Having shown that eq 1 provides reliable predictions of the maximum stacking interaction of druglike heterocycles with the nucleobases, we evaluated the stacking potential of a larger set of heterocycles. Figure 9 shows stacking interactions predicted using eq 1 based on the DFT-computed ESP_max_ values from Bootsma et al.^73^ for a set of 1854 heterocycles from the VEHICLe database of Pitt et al.^83^ These include both previously synthesized heterocycles and those that have not been made but are predicted to be synthetically tractable.^83^ The data are available in the SI and should serve as a source of druglike heterocycles offering a wide range of stacking interactions with nucleobases. This set of heterocycles exhibits similar trends in predicted maximum stacking interaction energies as the 54 heterocycles shown in Figure 5. For example, for each nucleobase, the potential stacking interactions span a wide range, with the mean stacking interaction following the trend A ≈ T ≈ U < C ≪ G. Using ESP_max_ values derived directly from SMILES^73^ rather than DFT computations results in very similar predictions (see SI Figure S6). Using these computationally inexpensive descriptors,^73^ one can use eq 1 to screen much larger sets of potential heterocycles with negligible computational cost.

### Predicting the Strength of Nonoptimal Stacking Interactions

**Figure 10:**
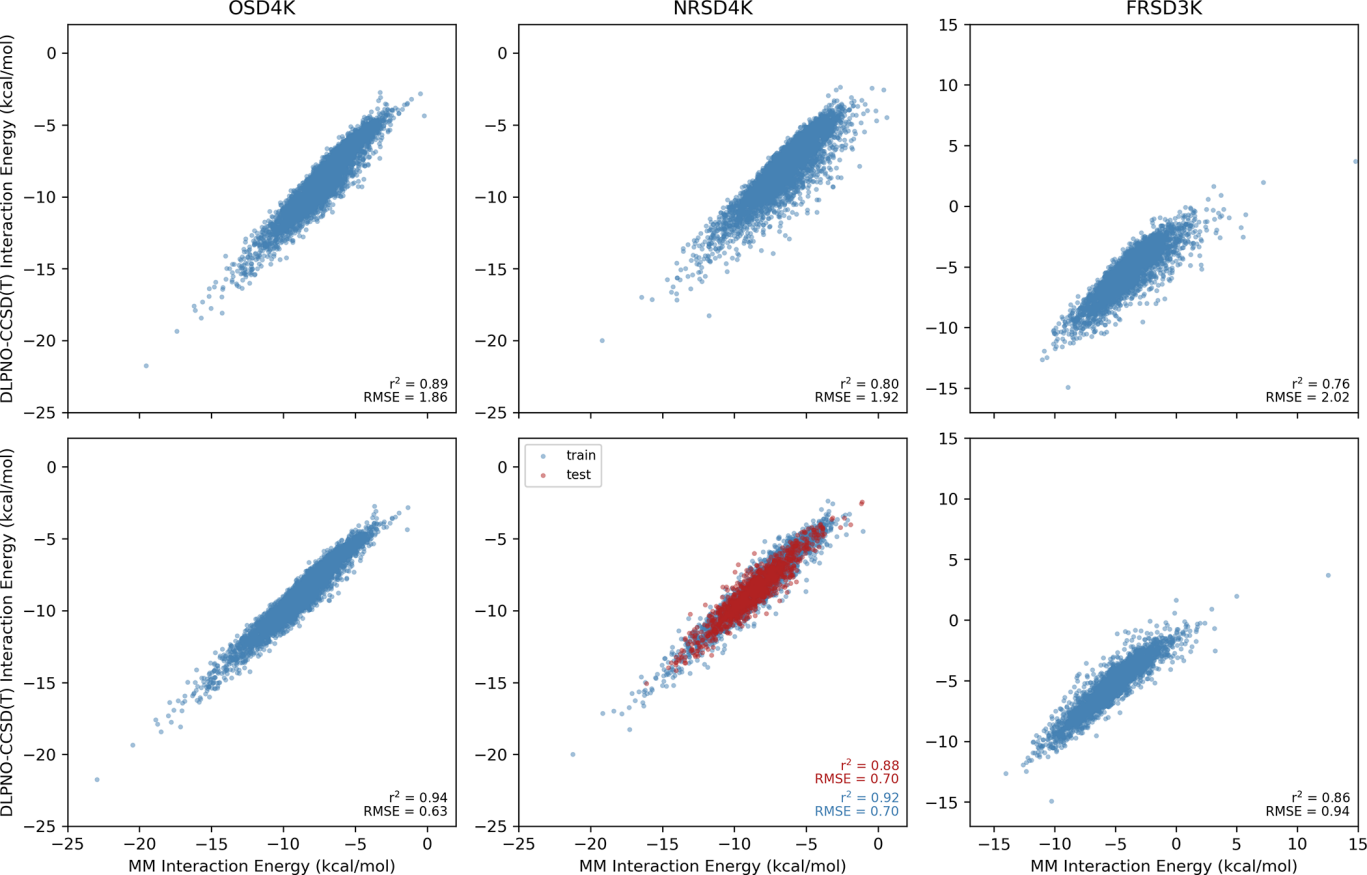
Interaction energies for the dimers in OSD4K, NRSD4K, and FRSD3K computed using GAFF2 (top) and scaled GAFF2 (bottom), both with HF/6-31G(d) RESP charges, vs DLPNO-CCSD(T) interaction energies.

While eq 1 can be used to predict the maximum possible stacking interaction of a druglike heterocycle with a given nucleobase, achieving the optimal stacking pose is rarely geometrically feasible within a given binding site. A means of predicting the interaction energy of a given stacking pose is necessary for structure-based design. While Ren and co-workers^47^ have developed a polarizable force field for nucleobase-heterocycle interactions, we sought to assess the ability of the noncovalent component of standard (fixed-charge) small-molecule MM force fields^84−87^ to predict these stacking interaction energies. RMSE and *r*^2^ values for GAFF, GAFF2, and Sage with various charge models applied to the three data sets are listed in Table 3. We note that MM parametrization implicitly accounts, in part, for solvation by water, yet we are comparing gas-phase interaction energies. Regardless, we expect that this comparison will provide insight into the performance of standard MM methods for nucleobase-heterocycle stacking interactions. Predicted interaction energies from GAFF2 [using HF/6-31G(d) RESP atomic charges] are representative and are plotted in Figure 10 versus the DLPNO-CCSD(T) values (plots for the other MM force fields can be found in SI Figures S7–S13). For the local minima in OSD4K, GAFF2 performs well, with predicted interaction energies strongly correlated with the QM reference values (*r*^2^ = 0.89) and a RMSE of 1.9 kcal/mol. This performance degrades slightly going first to NRSD4K and then to fully random stacked dimers (FRSD3K), for which *r*^2^ = 0.76 and RMSE 2.0 kcal/mol. This trend likely reflects the balance of attractive and repulsive interactions at the local stacked minima in OSD4K, which would lead to a greater cancelation of errors in the MM predictions that does not occur for the more varied geometries in NRSD4K and FRSD3K. The RMSE and correlation coefficient are improved slightly by using more expensive ωB97X-D/def2-TZVP RESP charges, while they degrade when using semiempirical AM1-BCC charges. For these commonly employed charges, the RMSE for GAFF2 is consistently above 2 kcal/mol. We note that GAFF2 paired with the recommended ABCG2 charges^87^ performs relatively poorly, compared to the other MM methods, for these gas-phase stacking interactions. Overall, results for GAFF and Sage are like those for GAFF2 (see SI Figures S10–S13); errors are around 2 kcal/mol with larger errors and more outliers for dimers farther removed from local energy minima.

**Table 3:** Performance of Standard and Scaled MM Potentials along with Optimized Scaling Constants (*C*_R_ and *C*_A_) for Heavy-Atom Pairs

				OSD4K		NRSD4K		FRSD3K	
standard MM				*r*^2^	RMSE	*r*^2^	RMSE	*r*^2^	RMSE
GAFF	AM1-BCC			0.80	1.85	0.67	2.02	0.66	2.08
	HF^b^			0.89	1.63	0.75	1.84	0.73	1.91
	DFT^c^			0.92	1.80	0.75	1.99	0.74	1.85
GAFF2	AM1-BCC			0.80	2.06	0.72	2.10	0.69	2.19
	ABCG2			0.68	2.54	0.61	2.56	0.58	2.62
	HF^b^			0.89	1.86	0.80	1.92	0.76	2.02
	DFT^c^			0.92	2.04	0.81	2.07	0.78	1.96
Sage	AM1-BCC			0.78	2.10	0.56	2.49	0.56	2.57

scaled MM^a^		*C*_R_	*C*_A_						
GAFF	AM1-BCC	0.78	0.99	0.84	0.99	0.82	0.99	0.79	1.10
	HF^b^	0.77	0.97	0.91	0.72	0.90	0.73	0.86	0.90
	DFT^c^	0.76	0.99	0.94	0.62	0.92	0.63	0.88	0.79
GAFF2	AM1-BCC	0.86	1.08	0.87	0.92	0.84	0.96	0.80	1.13
	ABCG2	0.89	1.13	0.79	1.27	0.77	1.28	0.73	1.35
	HF^b^	0.85	1.06	0.94	0.63	0.91	0.70	0.86	0.94
	DFT^c^	0.84	1.08	0.95	0.54	0.93	0.61	0.88	0.82
Sage	AM1-BCC	0.67	0.93	0.84	0.99	0.83	0.99	0.78	1.14

a For nonheavy-atom pairs, *C*_R_ = *C*_A_ = 1.

b RESP charges computed at the HF/6-31G(d) level of theory.

c RESP charges computed at the ωB97X-D/def2-TZVP level of theory.

To assess whether these MM force fields could be altered to provide more reliable predictions of stacking interactions across different stacking poses, we introduced two atom-type-independent parameters (*C*_R_ and *C*_A_) that scale the repulsive and attractive components of the intermolecular vdW terms of the interaction for pairs of heavy atoms. For each force field/atomic charge model, values of *C*_R_ and *C*_A_ were fit to minimize the root-mean-squared error in the predicted interaction energies for the training set (**1**–**40**) using data from NRSD4K. The optimized parameters are found in Table 3; predictions from a scaled GAFF2 potential for the heterocycle dimers in all three data sets are shown in the bottom panels of Figure 10. For each data set, there is a significant reduction in the RMSE, from close to 2 kcal/mol down to 0.63, 0.70, and 0.94 kcal/mol for OSD4K, NRSD4K, and FRSD3K, respectively, with much stronger correlations as well. This simple alteration ameliorates the tendency of GAFF2 to underbind stacked dimers while also drastically reducing the number of outliers, though some do remain. The scaling provides similar improvements for difference charge models and for GAFF and Sage (see Table 3 and SI Figures S10–S13). Perhaps most importantly from a practical standpoint, by scaling the vdW interaction, the errors in MM-predicted stacking interaction energies are reduced to less than 1 kcal/mol even when using AM1-BCC atomic charges. We note, however, that even with this scaling, the RMSE for GAFF2 paired with ABCG2 charges^87^ remains at 1.3 kcal/mol.

Obviously, this simple scaling of the terms in the vdW potential does not fix the underlying deficiencies of fixed-charged MM methods or the fact that they neglect the charge penetration effects that are vital for robust stacking predictions.^47,88,89^ Instead, it provides a pragmatic means of obtaining improved stacking interaction energies from existing computational workflows while not altering the nonbonded terms involving nonaromatic molecular components. Of course, there are other partial charges that could be employed instead of the standard RESP charges; some of these could provide more accurate stacking predictions when paired with GAFF, GAFF2, or Sage potentials. To this end, we note that the scaling factors provided in Table 3 are specific to the source of charges listed, because the scaling of the vdW parameters is, in part, compensating for deficiencies in the electrostatic term.

### Stacking Contributions to the Binding of Ribocil

Ribocil is a selective modulator of bacterial riboflavin riboswitches identified by Roemer et al.^7^ through a phenotypic screen of compounds that exhibited antibacterial activity. An X-ray crystal structure^7^ of ribocil bound to the flavin mononucleotide (FMN) riboswitch of *Fusobacterium nucleatum* (*F. nucleatum*) revealed that the exceptionally strong binding (*K*_d_ = 6.6 ± 2 nM) is due primarily to interactions of the three aromatic heterocyclic components with nucleobases. The central ring stacks with A85, whereas the terminal pyrimidyl group stacks with G62. The thiophene engages in both stacking interactions with A48 and an edge-to-face interaction with A49. To understand the contributions of these stacking interactions to the overall binding, we quantified the stacking in the binding pose and compared these to the model stacked dimers described above.

**Figure 11:**
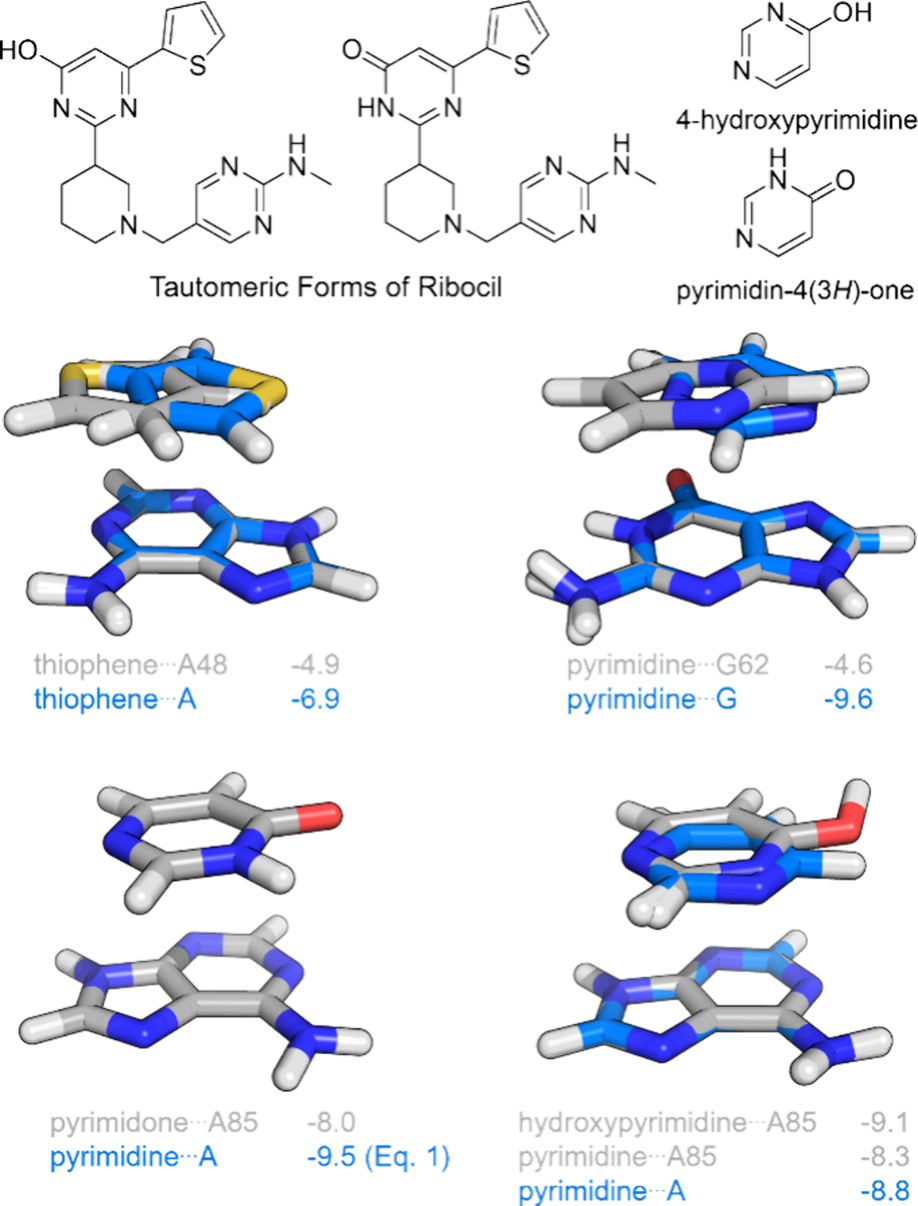
Comparison of stacking interactions (in kcal/mol) in the binding of ribocil to the FMN riboswitch (gray) vs the corresponding global energy minimum stacked dimers (blue).

Figure 11 shows the stacked dimer of thiophene with A48 superimposed over the global minimum energy dimer of thiophene (**3**) with adenine. The position of the ring centroid over A48 is similar to that in the global minimum stacked dimer; however, the ring is flipped 180° around C_2_. This orientation matches a local energy minimum about 1 kcal/mol higher in energy than the global minimum. Consequently, the thiophene···A48 stacking interaction (−4.9 kcal/mol) is about 70% of the maximum possible thiophene-adenine stacking interaction (−6.9 kcal/mol). This provides a practical reminder that stacking interactions in ligand binding sites often must compete with other noncovalent interactions. In this case, the thiophene is rotated 180° to engage in more favorable edge-to-face interactions with A49, which incurs a slight cost in terms of the stacking interaction with A48.

There is some uncertainty regarding the tautomeric form of the central arene ring in ribocil (see Figure 11), because 4-hydroxypyrimidine and its pyrimidone tautomer [pyrimidin-4(3*H*)-one] are essentially isoenergetic.^90^ Roemer et al.^91^ suggested that the pyrimidone tautomer was more likely given the presence of H-bonds to the 2’OH of A48 and exocyclic NH_2_ of A99. A model of the interaction between these two tautomeric forms and binding site functional groups indicates that while H-bonding favors the pyrimidone form, stacking is more favorable for the hydroxypyrimidine tautomer (see SI Figure S14). However, the OH conformation of hydroxypyrimidine required to engage in H-bonding interactions with the OH group of A48 and NH_2_ of A99 is nearly 6 kcal/mol higher in energy than the fully relaxed structure. As such, computations support Roemer’s suggestion that the pyrimidone form is more likely. Regardless, we quantified the stacking of both tautomeric forms with A85. The stacking interaction energy for the hydroxypyrimidine form (in a conformation consistent with H-bonding to the 2’OH of A48 and exocyclic NH_2_ of A99) is −9.1 kcal/mol, which is considerably more favorable than that of the pyrimidone tautomer (−8.0 kcal/mol). Pyrimidin-4(3*H*)-one is not included in either our training set of test set, so we do not have a global minimum energy stacked dimer geometry to compare. However, eq 1 predicts a maximum possible stacking interaction between pyrimidin-4(3*H*)-one and adenine of −9.5 kcal/mol. That is, the position of the pyrimidone ring in ribocil delivers a stacking interaction exceeding 80% of the estimated maximum value. For the pyrimidyl form, the position and orientation of hydroxypyridine relative to A85 are remarkably close to the global minimum energy pyrimidine···adenine dimer. In fact, stripping away the hydroxy group, the pyrimidine···A85 interaction energy (−8.3 kcal/mol) is 94% the strength of that in the global minimum energy pyrimidine···A geometry (−8.8 kcal/mol). In other words, if the dominant tautomer is the pyrimidyl form, then the binding pose of ribocil achieves essentially optimal stacking for this central ring. Even if it is the pyrimidonyl tautomer, the strength of the stacking interaction is close to optimal.

The strong stacking of thiophene and the central pyrimidine/pyrimidone rings can be contrasted with that of the terminal pyrimidine (*N*-methylpyrimidin-2-amine). It is obvious that this stacking geometry does not correspond to any local energy minimum, because the ring centroid of the pyrimidine is located directly over the centroid of the six-membered ring of guanine, whereas the data in Figure 6 show that minimum energy geometries exhibit ring centroids located over atoms/bonds of guanine. Indeed, the stacking interaction between pyrimidine and G62 (−4.6 kcal/mol) is less than half of the maximum possible stacking for this dimer (−9.6 kcal/mol). In other words, with regard to stacking interactions there is considerable room for improvement in terms of the position and orientation of the terminal pyrimidyl ring relative to G62.

### Summary and Concluding Remarks

Stacking interactions can contribute significantly to the binding of small molecules to RNA, and learning to harness the power of these interactions will likely prove important in the development of RNA-binding ligands.^8−19^ To this end, we analyzed stacking interactions between 54 druglike heterocycles and the five natural nucleobases based on three new data sets of optimized stacked dimers, random geometries near these optimized stacked dimers, and a set of random but reasonable stacked dimers. These data provide key insights into heterocycle-nucleobase stacking interactions. First, gas-phase nucleobase-heterocycle stacking interactions span a considerable range, suggesting that heterocycle choice can exert a significant influence on ligand-nucleobase binding affinities. Critically, for many heterocycles, the local energy minima for stacked dimers with each nucleobase exhibit a wide range of interaction energies, highlighting the importance of achieving optimal orientations of each heterocycle relative to a given nucleobase.^35^ Trends in stacking interactions are similar to those for stacking with aromatic amino acids, meaning that the guidelines from Bootsma et al.^38^ are also applicable to stacking with nucleobases. Second, we identify heterocycles for which stacking with a nucleobase is predicted to qualitatively alter the tautomeric equilibrium. Third, the optimized stacked dimer geometries cluster around a discrete set of stacking loci that are characteristic of each nucleobase. These stacking loci occur primarily over *N* atoms but also select C—C bonds and provide a small set of geometric targets above each nucleobase where stacking is expected to be most favorable. Fourth, the maximum stacking interaction of a given heterocycle-nucleobase pair can be predicted reliably using a simple model based on either DFT-computed descriptors^54^ or those derived directly from SMILES,^73^ allowing for the rapid screening of large libraries of heterocycles with regard to their nucleobase stacking proclivities. Finally, minor modifications to standard MM force fields can halve mean errors in predicted stacking energies, providing a pragmatic means of extracting more reliable stacking energies from existing computational workflows.

As always, these computational results come with many caveats. For instance, these data are based on stacking interactions with a single nucleobase, not the hydrogen-bonded nucleobase pairs in many RNA-binding pockets and consider idealized parallel stacking configurations that are unlikely to be fully representative of realistic stacking poses. Moreover, these gas-phase energy computations neglect not only entropic effects but also the impact of the dielectric environment of the RNA-binding pocket and the cost of desolvation that will accompany binding. As such, the practical use of these data and associated predictive model will require, at a minimum, separate consideration of desolvation costs. Previous data from Bootsma et al.^38^ for stacking interactions of druglike heterocycles with aromatic amino acid side chains suggest that the overall trends in binding energies will be similar in protein-like dielectric environments, but the range of values will be considerably smaller. However, Liedl et al.^79,92^ have recently demonstrated that solvent effects can impact both the strength and geometry of stacking interactions and that increased desolvation costs often overshadow any gain in stacking interaction energies,^93^ casting some doubt on the prudence of trying to enhance binding through stronger stacking interactions. Ultimately, application of GIST or similar approaches^94,95^ to the stacked heterocycle-nucleobase dimers discussed above will be critical to fully understand the interplay of solvent effects and stacking interactions. Despite these limitations, our hope is that these data and the associated predictive model, as well as the scaling constants for popular MM potentials, will prove useful as we advance toward the development of RNA-targeting small molecules.^8−19^

To this end, the results presented above will need to be validated experimentally. Togo et al.^35^ recently demonstrated the utility of a protein–ligand system that exhibits a conserved binding pose featuring stacking interactions between a pendant aryl group and a pair of Tyr residues as a means of experimentally probing the strength of heterocycle-Tyr stacking interactions. The binding of ribocin by the FMN riboswitch (Figure 1a)^7,91^ could potentially provide an analogous platform for quantifying stacking interactions of heterocycles with guanine within a realistic biological environment. Roemer et al.^91^ showed that the ribocil binding pose is insensitive to changes of the pendant pyrimidyl group, in which case variations in experimental binding constants for congeners of ribocil with varying heterocycles at this position should correlate with differences in stacking interactions between the heterocycle and guanine.

## Data Availability

All data, including molecular structures, are available in the Supporting Information.

## Supporting Information Available

Cartesian coordinates of all computed dimers (FRSD3K) (XYZ)
Cartesian coordinates of all computed dimers (NRSD4K) (XYZ)
Cartesian coordinates of all computed dimers (OSD4K) (XYZ)
Electronic energies and SAPT data (XLSX)
Additional tables, figures, and computational details (PDF)

## Supplementary Material

ci4c02420_si_001.xyz

ci4c02420_si_002.xyz

ci4c02420_si_003.xyz
