# Supplementary material for: Stacking Interactions of Druglike Heterocycles with Nucleobases
Source: J Chem Inf Model. 2025 Mar 27;65(7):3502–16. doi: 10.1021/acs.jcim.4c02420 (PMC12004538; doi:10.1021/acs.jcim.4c02420)
Supplement: Supplementary file 5 — ci4c02420_si_005.pdf [file ci4c02420_si_005.pdf]

Supporting Information

Stacking Interactions of Drug-Like Heterocycles with Nucleobases

Audrey V. Conner, Lauren M. Kim, Patrick A. Fagan, Drew P. Harding, and Steven E. Wheeler*

Department of Chemistry, University of Georgia, Athens, GA 30602
E-mail: swheele2@uga.edu

Contents pp.

Table S1. Binding energies (in kcal/mol) for global minimum stacked dimers of heterocycles **1**-**54** with the nucleobases. S2

Table S2. Mean binding energies (in kcal/mol) across all local minimum stacked dimers of heterocycles **1**-**54** with the nucleobases. S3

T**able S3.** Correlation coefficients of maximum and mean binding energies for a given heterocycle across the five nucleobases. S4

Table S4. AM1-BCC and RESP charges for the heteroatom in selected molecules based on HF/6-31G(d) and wB97XD/def2-TZVP. S4

Figure S1. Predicted interaction energies from Eq. 2 vs total SAPT interaction energies for all local minima in OSD4K. S5

Figure S2. Total SAPT interaction energy vs E_{exch} and not E_{exch} (top) and E_{ind} and not E_{ind} (bottom). S6

Figure S3. Computed binding energies for the global minimum energy dimers in OSD4K vs binding energies predicted using Eq. 1 with DFT-based descriptors for the training set (top panels) and test set (bottom panels), separated by nucleobase. S7

Figure S4. Computed binding energies for the global minimum energy dimers in OSD4K vs binding energies for **1**-**54** predicted using Eq. 1 with SMILES-based descriptors. S8

Figure S5. Computed binding energies for the global minimum energy dimers in OSD4K vs binding energies predicted using Eq. 3. (top) training set (**1**-**40**) predicted using DFT-based descriptors; (bottom) test set (**41**-**54**) predicted using DFT-based descriptors. S9

Figure S6. Predicted maximum stacking energies for each nucleobase with the set of 1854 heterocycles predicted from Eq. 1 using SMILES-based ESP_{max} values vs DFT-computed ESP_{max} values. S10

Figures S7-S13. Interaction energies for the dimers in OSD4K, NRSD4K, and FRSD3K computed using unscaled (top) and scaled (bottom) MM methods with different charge models, vs DLPNO-CCSD(T) interaction energies. S11

Figure S14. Model of interactions for tautomers of ribosyl. S14

Computational Details S15

References S17

Table S1. Binding energies (in kcal/mol) for global minimum stacked dimers of heterocycles **1-54** with the nucleobases.

	A	C	G	T	U		A	C	G	T	U
**1**	-7.9	-8.1	-11.2	-7.3	-7.2	**30**	-10.9	-12.1	-15.6	-11.0	-10.7
**2**	-6.1	-5.8	-8.3	-6.2	-5.7	**31**	-12.6	-14.3	-18.0	-11.4	-10.2
**3**	-6.6	-6.0	-8.7	-6.3	-6.0	**32**	-11.0	-12.2	-15.5	-10.2	-9.5
**4**	-7.5	-7.1	-10.3	-6.9	-6.7	**33**	-13.5	-13.7	-16.9	-11.9	-11.4
**5**	-7.5	-6.3	-9.5	-6.7	-6.3	**34**	-13.5	-13.9	-17.4	-13.3	-12.8
**6**	-7.2	-6.9	-10.6	-6.6	-6.5	**35**	-12.7	-13.0	-16.6	-11.7	-10.8
**7**	-7.4	-6.6	-9.6	-7.0	-6.8	**36**	-15.7	-16.7	-22.3	-13.0	-12.8
**8**	-7.9	-7.9	-10.8	-7.6	-7.6	**37**	-10.4	-10.2	-13.8	-10.2	-9.8
**9**	-8.6	-9.4	-13.3	-7.9	-7.9	**38**	-11.9	-11.2	-15.5	-10.6	-10.5
**10**	-8.7	-9.2	-11.5	-7.5	-7.5	**39**	-11.0	-10.7	-13.9	-10.4	-9.9
**11**	-9.0	-9.7	-13.4	-8.7	-8.4	**40**	-12.4	-14.6	-18.1	-12.4	-12.2
**12**	-8.2	-6.5	-9.3	-7.3	-7.1	**41**	-10.3	-11.3	-14.6	-8.8	-8.6
**13**	-8.1	-8.6	-11.1	-8.2	-8.0	**42**	-8.6	-11.6	-13.2	-8.1	-7.5
**14**	-8.6	-8.2	-10.9	-8.3	-7.8	**43**	-7.1	-6.9	-9.0	-6.6	-6.4
**15**	-7.0	-6.3	-9.2	-6.8	-6.4	**44**	-8.0	-7.6	-10.3	-7.4	-6.9
**16**	-8.1	-7.3	-11.2	-7.2	-7.0	**45**	-10.7	-10.6	-12.7	-10.3	-10.1
**17**	-8.6	-7.3	-10.1	-7.3	-7.1	**46**	-11.7	-11.0	-15.3	-11.3	-10.9
**18**	-8.6	-7.4	-10.3	-6.7	-5.8	**47**	-10.7	-10.2	-13.6	-10.1	-9.6
**19**	-9.9	-9.5	-12.9	-8.4	-8.0	**48**	-10.1	-9.7	-12.8	-9.6	-9.2
**20**	-8.5	-7.3	-9.4	-6.8	-6.2	**49**	-10.5	-9.3	-12.6	-10.0	-9.6
**21**	-9.7	-9.9	-12.0	-8.0	-7.8	**50**	-11.4	-11.2	-14.6	-10.3	-9.5
**22**	-9.6	-10.9	-13.1	-8.6	-7.9	**51**	-11.4	-11.1	-14.2	-10.2	-9.4
**23**	-9.1	-8.0	-10.8	-7.2	-6.8	**52**	-12.9	-12.4	-15.6	-11.6	-11.0
**24**	-8.9	-9.6	-10.6	-8.0	-7.4	**53**	-11.3	-11.1	-14.8	-10.7	-10.3
**25**	-9.9	-11.6	-13.2	-8.5	-8.0	**54**	-12.2	-12.4	-16.8	-11.7	-11.2
**26**	-12.0	-12.0	-15.4	-11.3	-10.7	Max.	-15.7	-16.7	-22.3	-13.3	-12.8
**27**	-11.0	-11.3	-14.5	-10.8	-10.4	Min.	-6.1	-5.8	-8.3	-6.2	-5.7
**28**	-11.6	-11.5	-15.3	-11.1	-10.7	Mean	-9.9	-9.9	-13.0	-9.1	-8.7
**29**	-11.0	-10.0	-13.4	-10.5	-9.9						

Table S2. Mean binding energies (in kcal/mol) across all local minimum stacked dimers of heterocycles **1**-**54** with the nucleobases.

	**A**	**C**	**G**	**T**	**U**		**A**	**C**	**G**	**T**	**U**
**1**	-6.7	-6.4	-8.6	-6.8	-6.7	**30**	-9.6	-8.6	-12.4	-8.8	-8.8
**2**	-5.4	-5.0	-7.1	-5.5	-5.4	**31**	-10.7	-10.5	-12.1	-9.2	-8.2
**3**	-5.8	-5.4	-7.7	-6.0	-5.8	**32**	-9.4	-7.6	-11.3	-8.4	-7.6
**4**	-5.8	-6.4	-8.4	-5.5	-5.8	**33**	-10.8	-11.1	-14.1	-9.5	-8.5
**5**	-5.5	-5.4	-7.7	-5.4	-5.5	**34**	-11.1	-12.1	-14.8	-9.4	-8.8
**6**	-6.0	-5.8	-8.3	-5.5	-6.0	**35**	-11.4	-9.8	-12.9	-9.3	-8.7
**7**	-5.9	-5.6	-8.1	-5.9	-5.9	**36**	-12.3	-11.5	-14.3	-10.9	-9.9
**8**	-6.6	-6.6	-9.0	-6.7	-6.6	**37**	-9.6	-8.9	-11.7	-8.5	-7.8
**9**	-6.8	-7.9	-9.8	-6.6	-6.8	**38**	-9.7	-10.4	-13.6	-9.2	-8.0
**10**	-6.8	-5.9	-8.9	-6.4	-6.8	**39**	-9.7	-8.4	-11.5	-8.1	-7.4
**11**	-7.9	-9.1	-10.8	-6.9	-7.9	**40**	-10.9	-10.7	-14.1	-9.1	-8.6
**12**	-6.1	-5.9	-8.2	-6.2	-6.1	**41**	-7.7	-10.1	-11.6	-7.8	-7.3
**13**	-6.7	-6.7	-9.0	-6.2	-6.7	**42**	-7.7	-9.0	-10.6	-6.4	-7.1
**14**	-6.8	-7.7	-9.8	-5.9	-6.8	**43**	-5.8	-5.7	-7.2	-5.2	-4.8
**15**	-6.7	-6.0	-8.4	-6.7	-6.7	**44**	-6.1	-6.8	-8.9	-5.3	-5.1
**16**	-6.9	-6.6	-9.3	-6.3	-6.9	**45**	-8.9	-8.4	-11.2	-8.6	-8.1
**17**	-6.9	-6.4	-9.8	-6.6	-6.9	**46**	-9.3	-9.9	-12.1	-8.7	-8.0
**18**	-7.1	-5.4	-7.8	-5.6	-7.1	**47**	-9.2	-8.1	-11.7	-9.0	-8.1
**19**	-8.0	-8.5	-11.5	-7.5	-8.0	**48**	-9.0	-7.9	-11.0	-8.2	-7.7
**20**	-7.6	-6.5	-8.6	-5.9	-7.6	**49**	-9.3	-8.8	-11.7	-9.1	-8.4
**21**	-7.9	-7.4	-9.9	-6.5	-7.9	**50**	-10.0	-8.8	-11.8	-8.5	-7.6
**22**	-8.6	-10.0	-11.7	-7.4	-8.6	**51**	-10.1	-8.8	-12.0	-8.2	-7.4
**23**	-7.8	-7.6	-9.7	-6.3	-7.8	**52**	-10.6	-9.4	-13.3	-9.4	-8.9
**24**	-7.8	-9.0	-9.2	-6.5	-7.8	**53**	-9.6	-9.7	-12.8	-8.6	-7.8
**25**	-8.3	-7.7	-10.6	-8.0	-8.3	**54**	-10.5	-10.5	-13.0	-8.7	-7.6
**26**	-9.9	-11.0	-13.4	-8.2	-9.9	Max.	-12.3	-12.1	-14.8	-10.9	-9.9
**27**	-9.4	-8.4	-11.3	-9.1	-9.4	Min.	-5.4	-5.0	-7.1	-5.2	-4.8
**28**	-9.0	-8.6	-11.2	-9.0	-9.0	Mean	-8.3	-8.1	-10.7	-7.5	-7.0
**29**	-9.0	-8.6	-11.4	-8.9	-9.0						

Table S3. Correlation coefficients of maximum and mean binding energies for a given heterocycle across the five nucleobases.

	A	C	G	T	U
	Maximum Binding Energy				
A	1.00	0.87	0.90	0.92	0.89
C		1.00	0.94	0.84	0.81
G			1.00	0.87	0.86
T				1.00	0.99
U					1.00
	Mean Binding Energy				
A	1.00	0.77	0.89	0.88	0.83
C		1.00	0.89	0.67	0.68
G			1.00	0.83	0.82
T				1.00	0.95
U					1.00

Table S4. AM1-BCC and RESP charges for the heteroatom in selected molecules based on HF/6-31G(d) and wB97X-D/def2-TZVP.

	**AM1-BCC**	**HF/6-31G(d)**	**wB97XD/def2-TZVP**
H_2O	-0.79	-0.81	-0.77
CH_3OH	-0.60	-0.67	-0.61
$(CH_3)_2O$	-0.42	-0.37	-0.29
Pyridine	-0.67	-0.67	-0.64
2-methylpyridine	-0.67	-0.74	-0.72
3-methylpyridine	-0.67	-0.65	-0.63
4-methylpyridine	-0.67	-0.73	-0.70

$$E_{int}^{pred} = 1.06E_{elec} - 1.46 \quad (2)$$

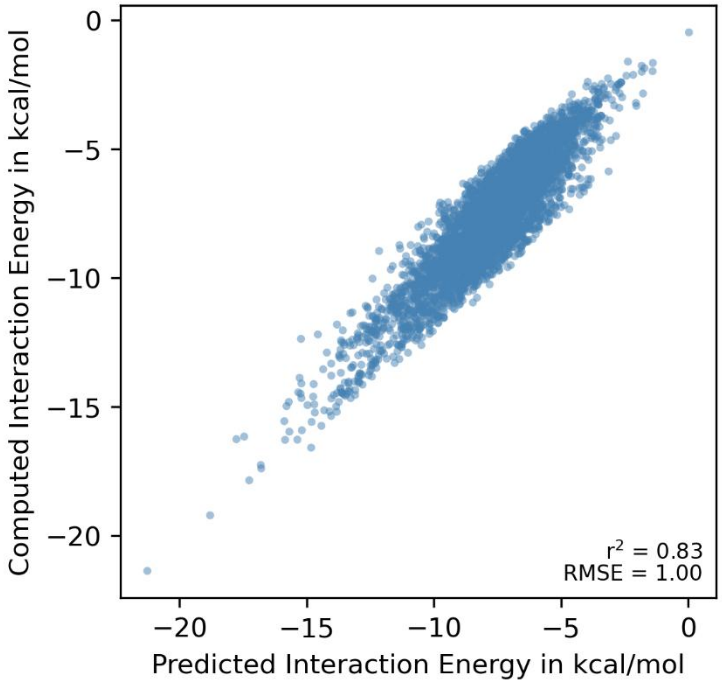

Figure S1. Predicted interaction energies from Eq. 2 (above) vs total SAPT interaction energies for all local minima in OSD4K.

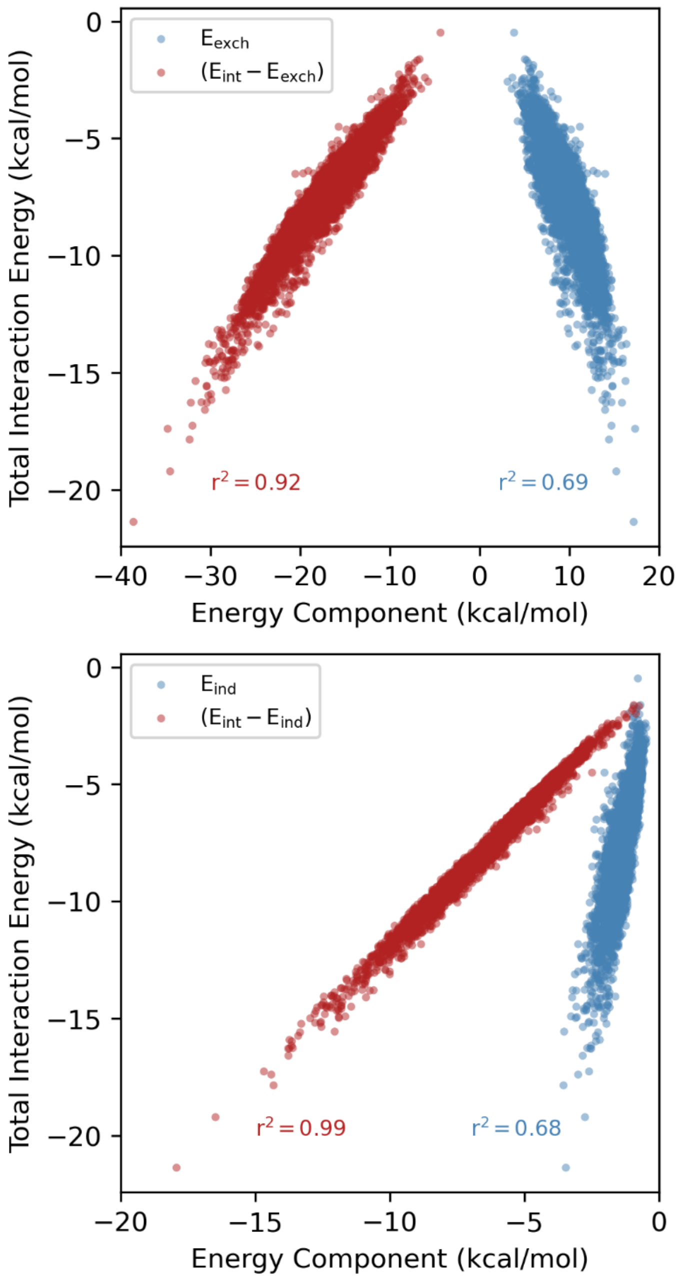

Figure S2. Total SAPT interaction energy vs E_{exch} and not E_{exch} (top) and E_{ind} and not E_{ind} (bottom).

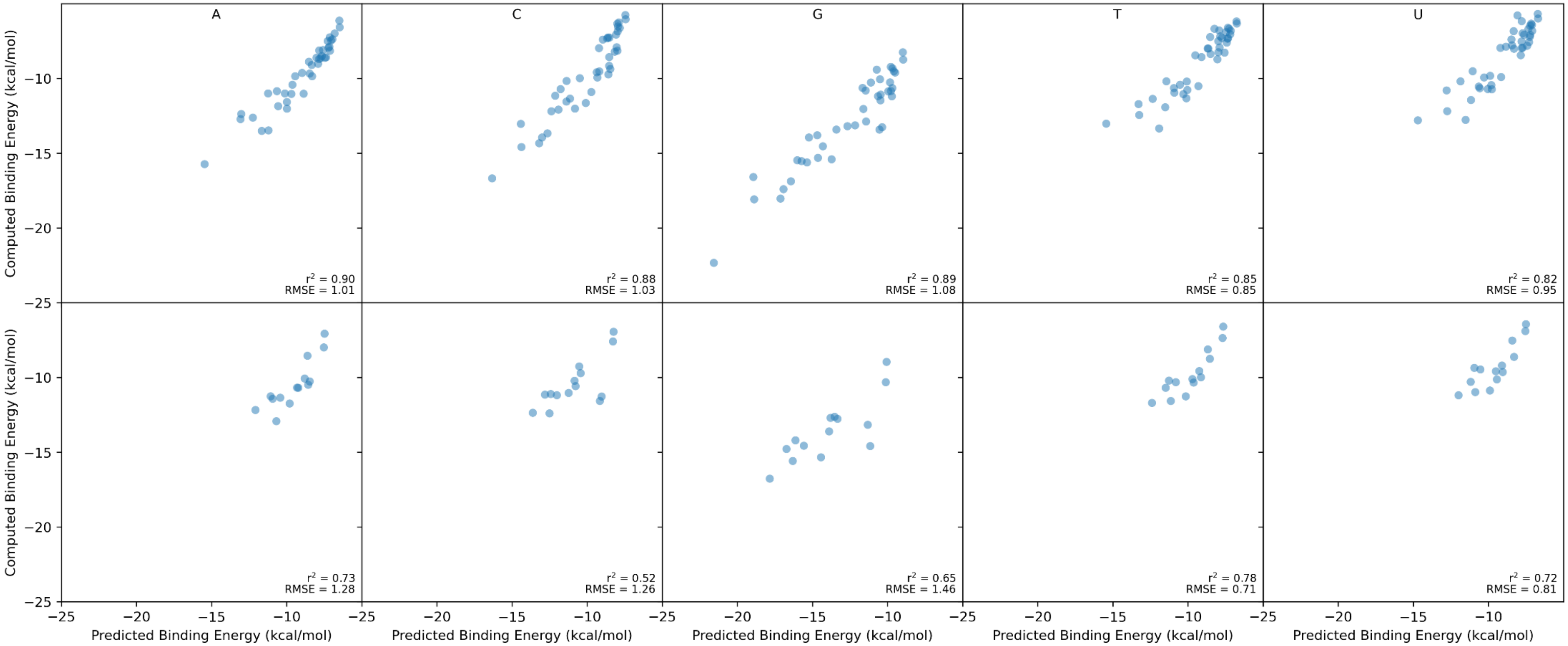

Figure S3. Computed binding energies for the global minimum energy dimers in OSD4K vs binding energies predicted using Eq. 1 (from the main text) with DFT-based descriptors for the training set (top panels) and test set (bottom panels), separated by nucleobase.

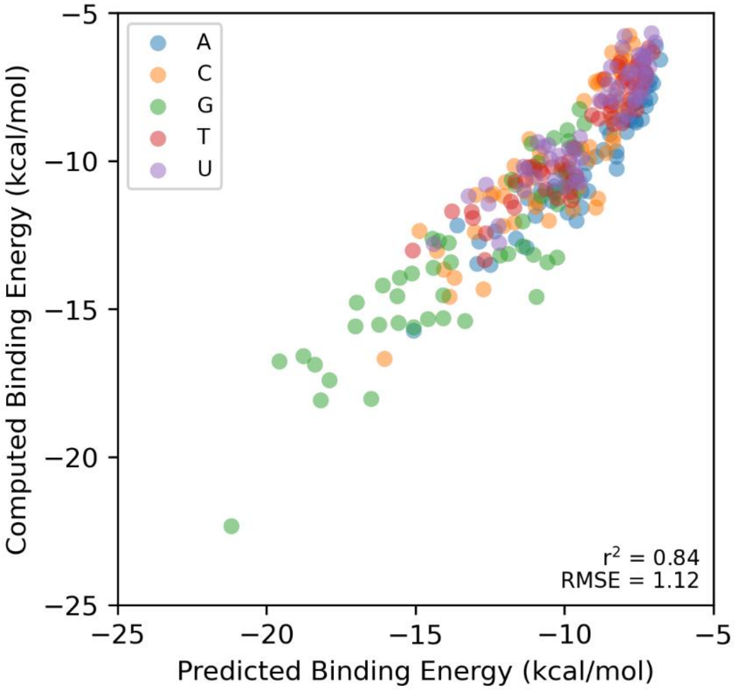

Figure S4. Computed binding energies for the global minimum energy dimers in OSD4K vs binding energies for **1**-**54** predicted using Eq. 1 (from the main text) with SMILES-based descriptors for the heterocycles.

$$E_{bind\,max}^{pred} = -0.0007\ N_{HA}^{Nuc} N_{HA}^{Het} (\mu^{Nuc} + \mu^{Het}) - 5.17 \quad (3)$$

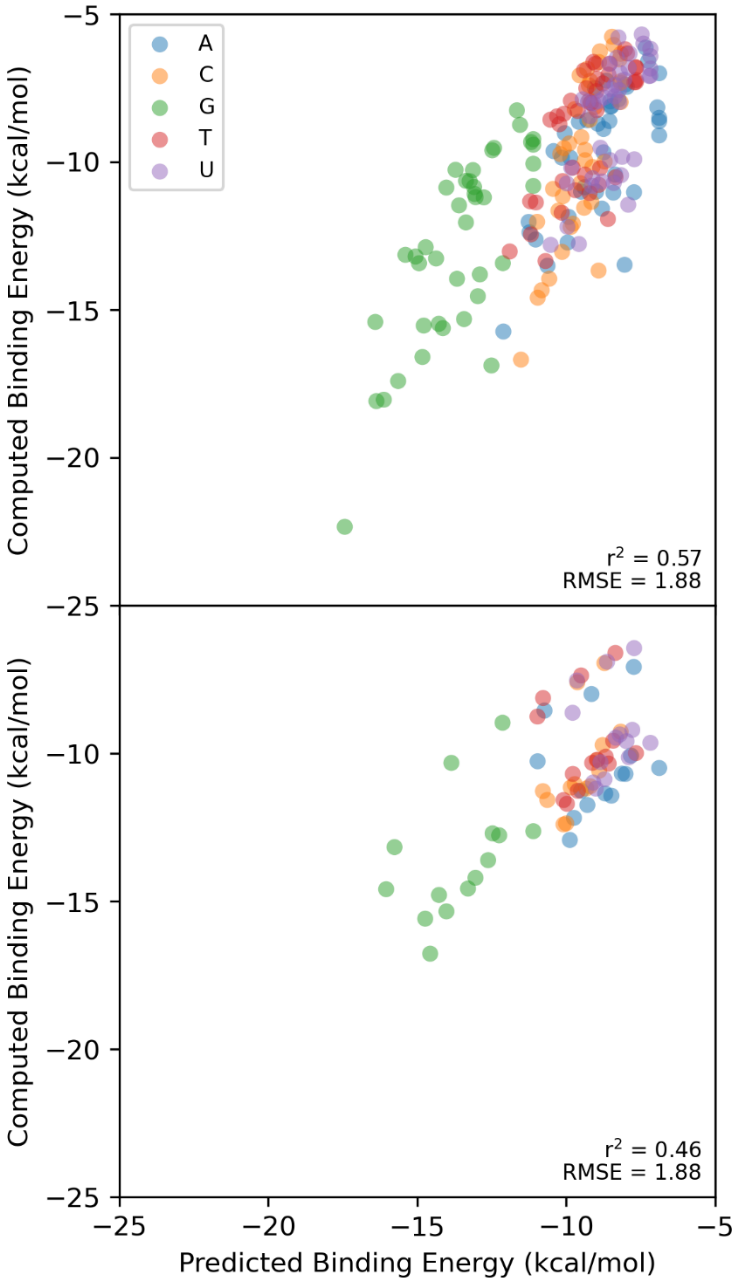

Figure S5. Computed binding energies for the global minimum energy dimers in OSD4K vs binding energies predicted using Eq. 3. (top) training set (**1**-**40**) predicted using DFT-based descriptors; (bottom) test set (**41**-**54**) predicted using DFT-based descriptors.

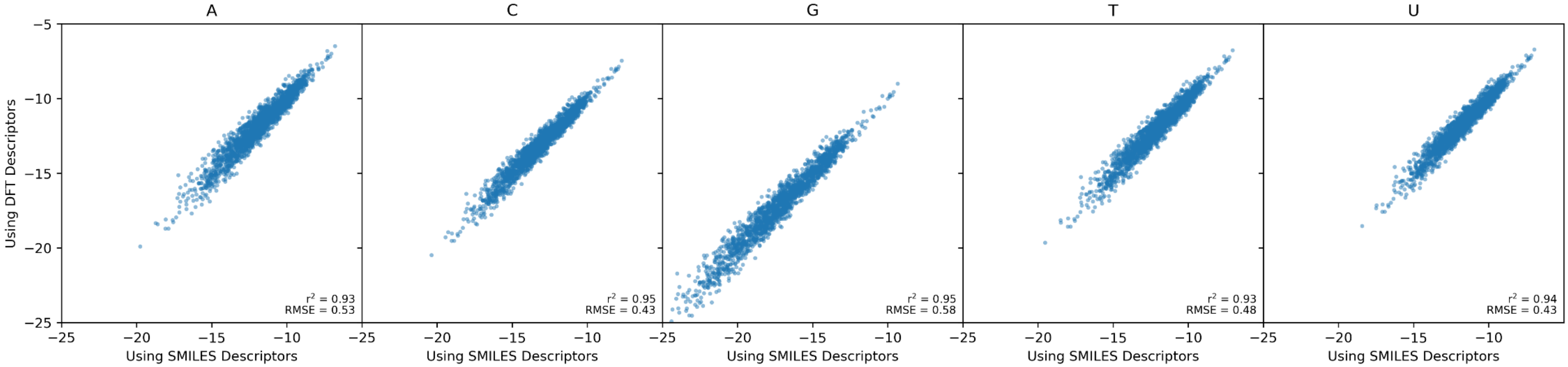

Figure S6. Predicted maximum stacking energies for each nucleobase with the set of 1854 heterocycles predicted from Eq. 1 (from the main text) using SMILES-based ESP_{max} values vs DFT-computed ESP_{max} values.

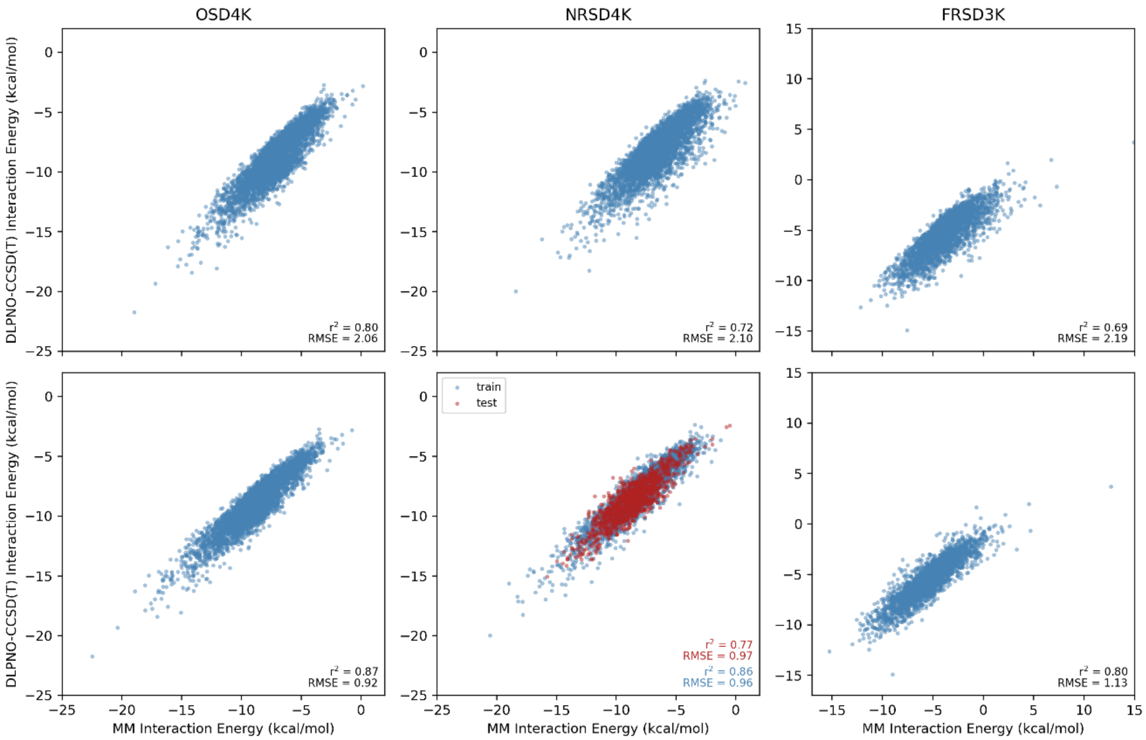

Figure S7. Interaction energies for the dimers in OSD4K, NRSD4K, and FRSD3K computed using GAFF2 (top) and scaled-GAFF2 (bottom), both with AM1-BCC charges, vs DLPNO-CCSD(T) interaction energies.

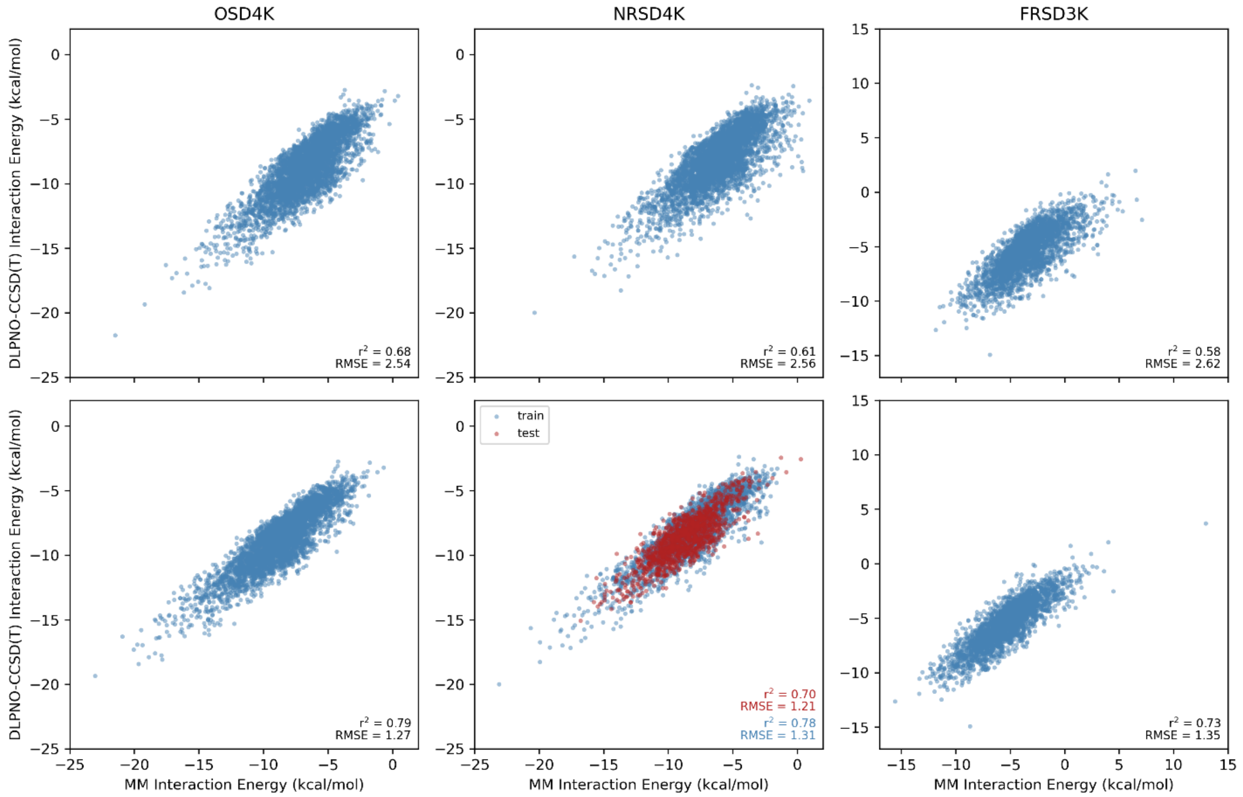

Figure S8. Interaction energies for the dimers in OSD4K, NRSD4K, and FRSD3K computed using GAFF2 (top) and scaled-GAFF2 (bottom), both with ABCG2 charges, vs DLPNO-CCSD(T) interaction energies.

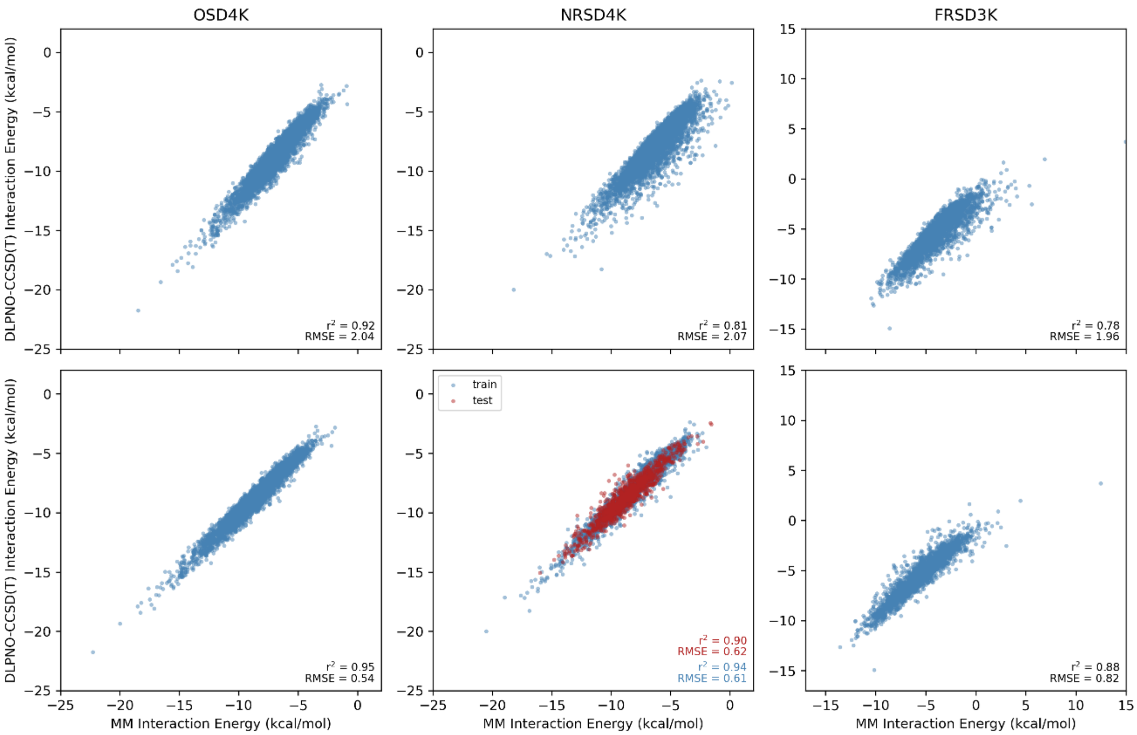

Figure S9. Interaction energies for the dimers in OSD4K, NRSD4K, and FRSD3K computed using GAFF2 (top) and scaled-GAFF2 (bottom), both with wB97X-D/def2-TZVP RESP charges, vs DLPNO-CCSD(T) interaction energies.

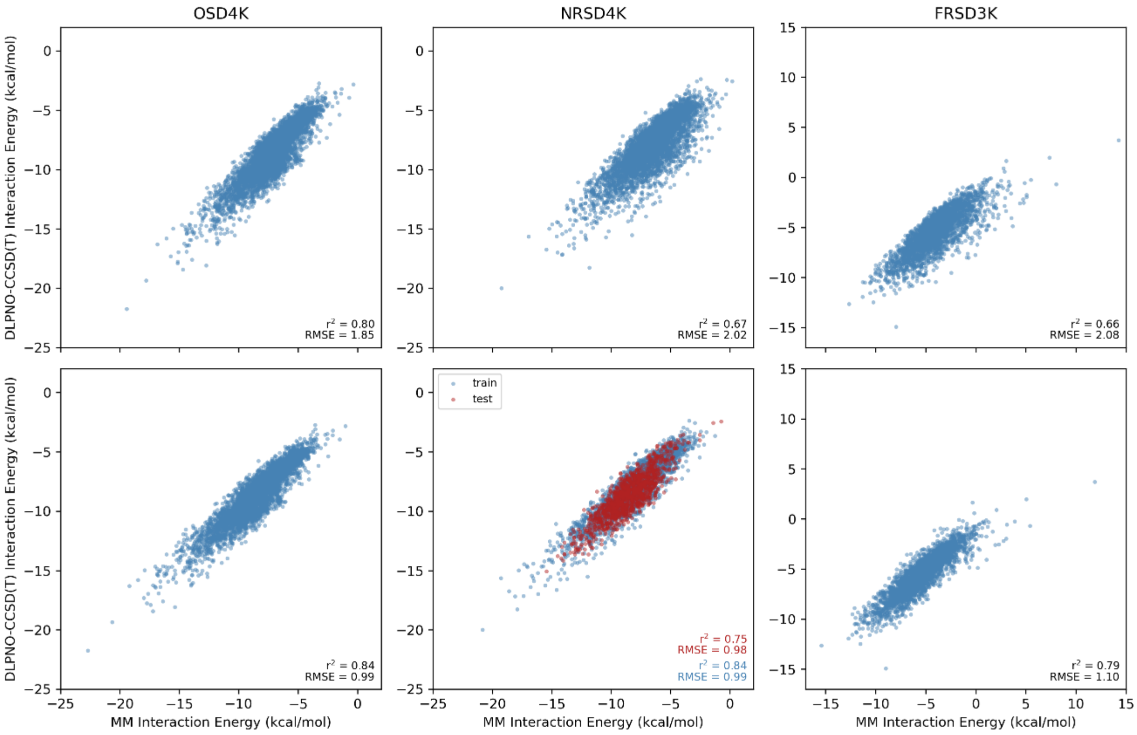

Figure S10. Interaction energies for the dimers in OSD4K, NRSD4K, and FRSD3K computed using GAFF (top) and scaled-GAFF (bottom), both with AM1-BCC charges, vs DLPNO-CCSD(T) interaction energies.

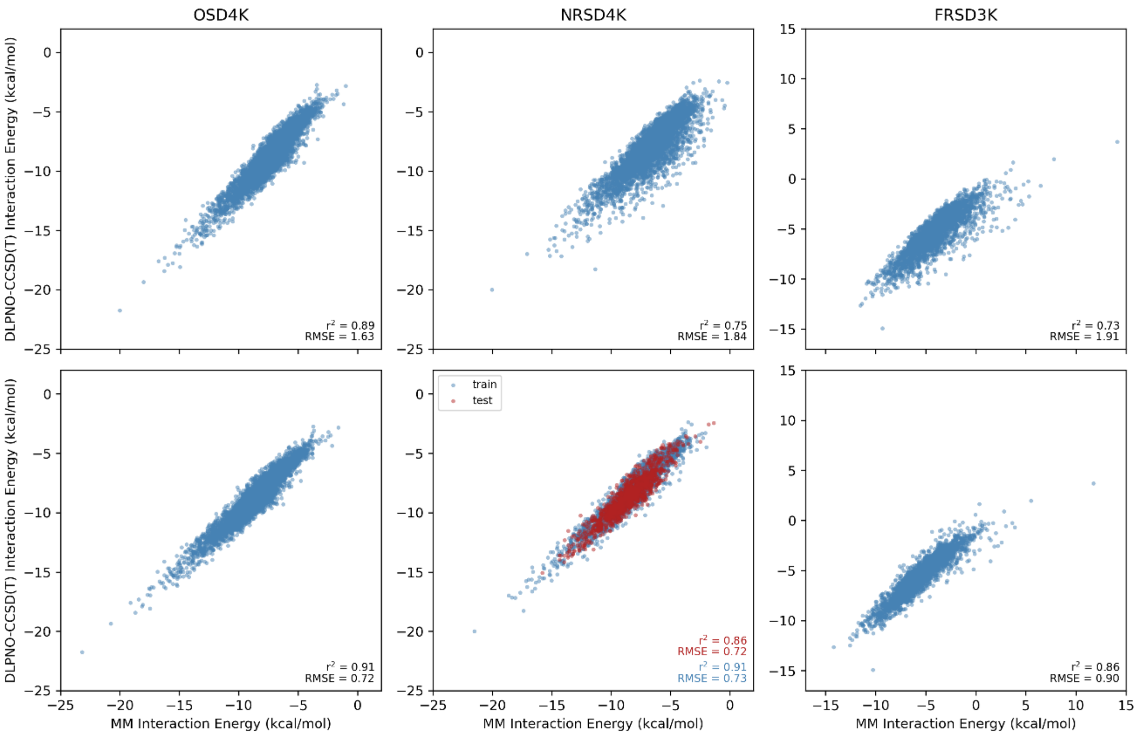

Figure S11. Interaction energies for the dimers in OSD4K, NRSD4K, and FRSD3K computed using GAFF (top) and scaled-GAFF (bottom), both with HF/6-31G(d) RESP charges, vs DLPNO-CCSD(T) interaction energies.

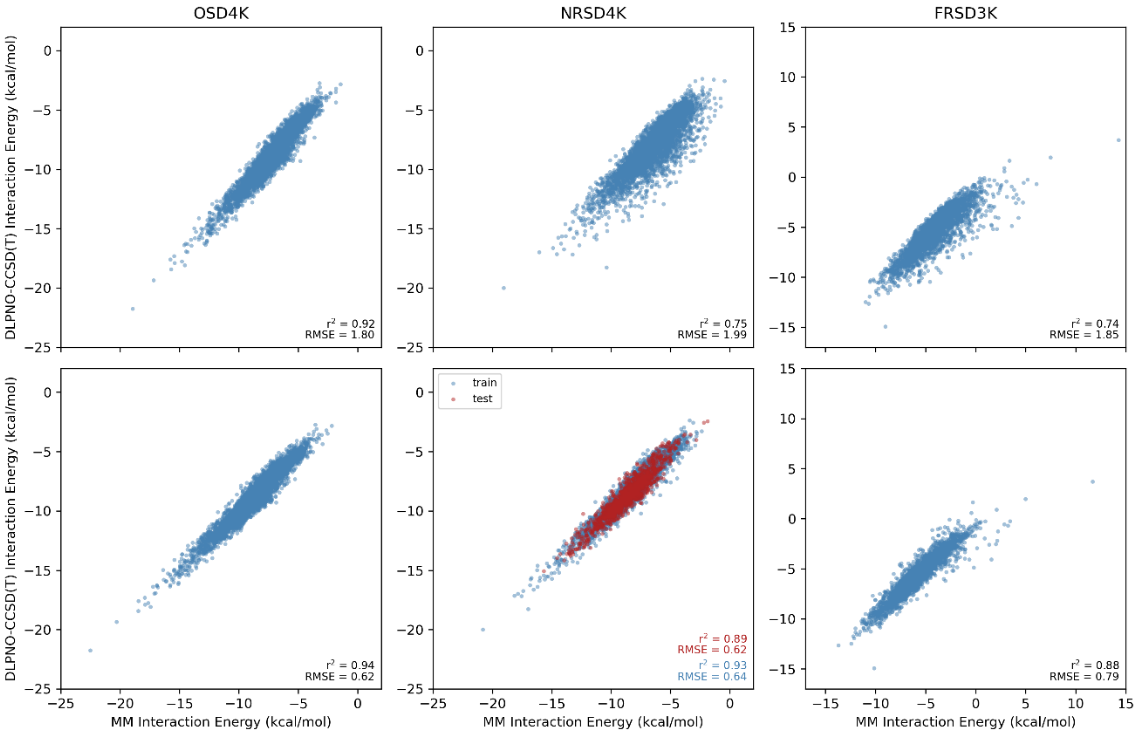

Figure S12. Interaction energies for the dimers in OSD4K, NRSD4K, and FRSD3K computed using GAFF (top) and scaled-GAFF (bottom), both with wB97X-D/def2-TZVP RESP charges, vs DLPNO-CCSD(T) interaction energies.

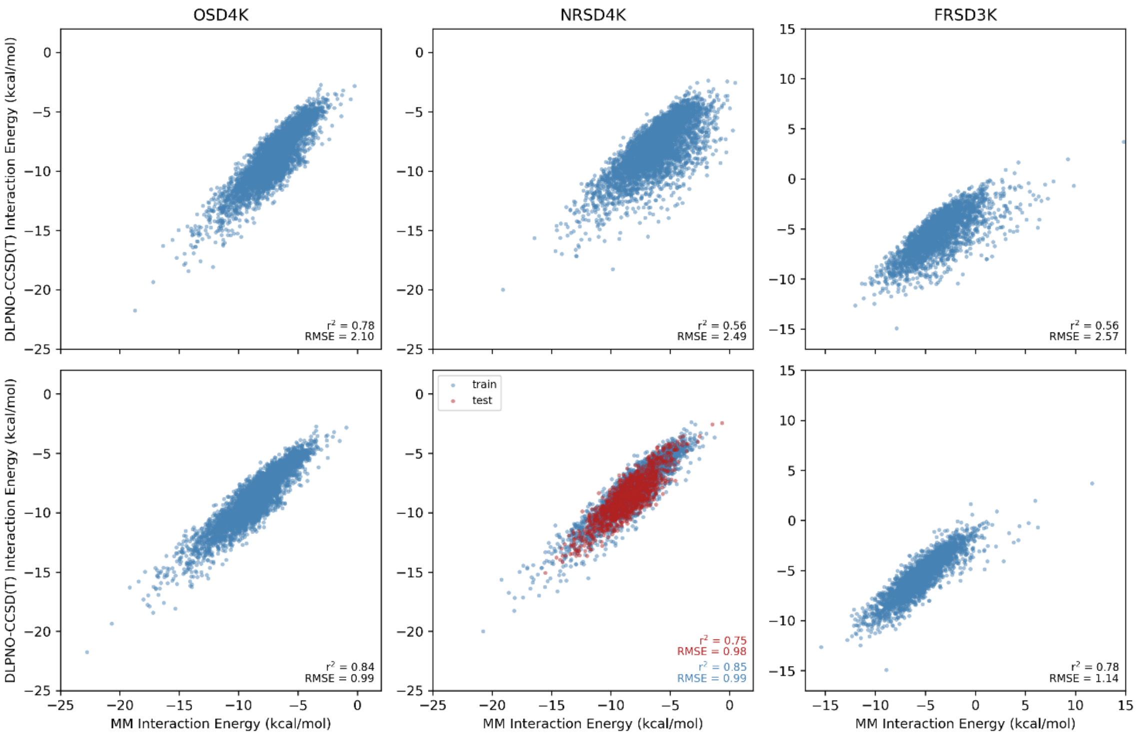

Figure S13. Interaction energies for the dimers in OSD4K, NRSD4K, and FRSD3K computed using Sage (top) and scaled-Sage (bottom), both with AM1-BCC charges, vs DLPNO-CCSD(T) interaction energies.

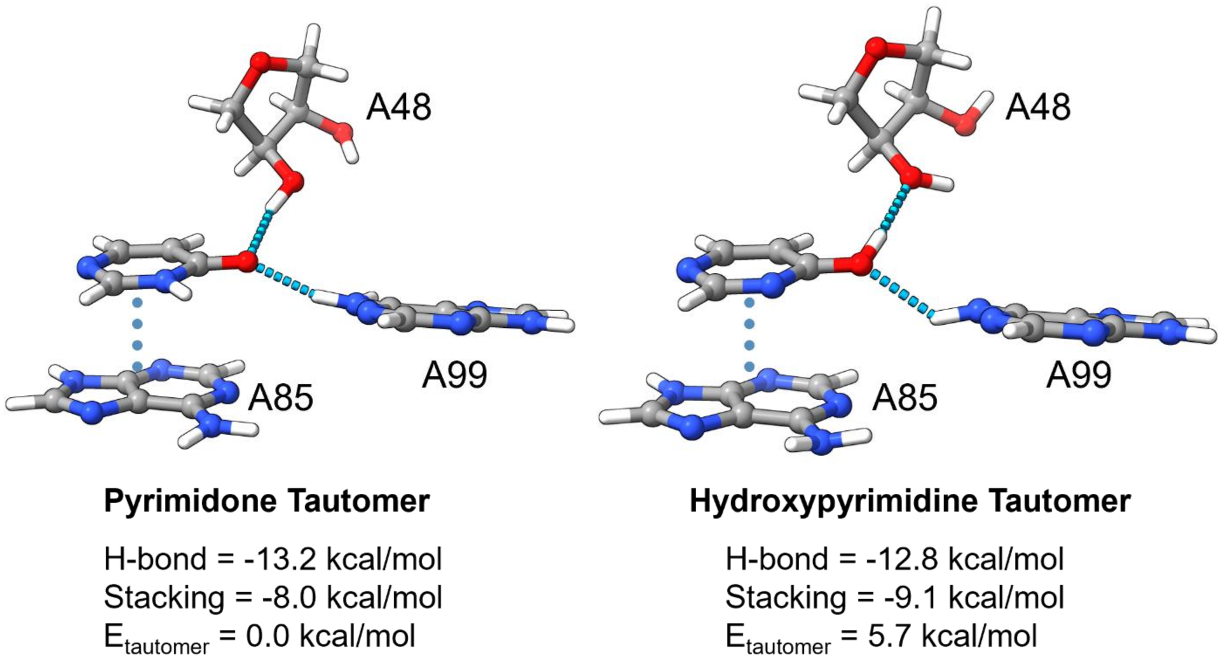

Figure S14. H-bonding and stacking interactions (kcal/mol) of pyrimidone and 5-hydroxypyrimidine. The relative energies of the tautomers ($E_{tautomer}$, in kcal/mol) is in the conformations shown. All energies computed at the DLPNO-CCSD(T)/def2-TZVP level of theory. H-atom positions optimized at the wB97X-D/def2-TZVP level, with heavy-atom positions from PDB 5C45.

Computational Details

Notes on SAPT0 Computations

We note that the induction energy in SAPT0 includes the δHF correction.

DLPNO-CCSD(T) Details

The DLPNO-CCSD(T) data presented in the main text used 'NormalPNO' cutoffs as defined in ORCA 4, which are as follows:

```
TCutMKN:          1.000e-03
TCutPNO:          3.330e-07
TCutPairs:        1.000e-04
TCutDO:           1.000e-02
```

Below, we also considered 'TightPNO' cutoffs for selected systems, which are

```
TCutMKN:          1.000e-03
TCutPNO:          1.000e-07
TCutPairs:        1.000e-05
TCutDO:           5.000e-03
```

To probe the convergence of the DLPNO-CCSD(T)/cc-pVTZ interaction energies with respect to basis set and PNO cutoffs, we considered a subset of 108 random stacked dimers by selecting one random dimer of adenine and cytosine with each of the 54 heterocycles. Interaction energies were computed using DLPNO-CCSD(T) with cc-pVQZ and aug-cc-pVTZ using NormalPNO cutoffs (see above) as well as cc-pVTZ with TightPNO cutoffs (see above). Data is provided in `ci4c02420_si_004.xls`.

The RMSE for DLPNO-CCSD(T)/cc-pVTZ/NormalPNO compared to DLPNO-CCSD(T)/aug-cc-pVTZ/NormalPNO and DLPNO-CCSD(T)/cc-pVTZ/TightPNO were 1.0 and 0.9 kcal/mol, respectively. However, these errors are strongly anti-correlated and cancel to a large degree. For instance, the RMSE for DLPNO-CCSD(T)/cc-pVTZ/NormalPNO compared to

DLPNO-CCSD(T)/aug-cc-pVTZ/TightPNO ≈ DLPNO-CCSD(T)/aug-cc-pVTZ/NormalPNO
+ [DLPNO-CCSD(T)/cc-pVTZ/TightPNO - DLPNO-CCSD(T)/cc-pVTZ/NormalPNO]

is only 0.2 kcal/mol. Further extending this *ad hoc* composite method to include basis set up to QZ:

DLPNO-CCSD(T)/aug-cc-pVQZ/TightPNO ≈ DLPNO-CCSD(T)/aug-cc-pVTZ/NormalPNO
+ [DLPNO-CCSD(T)/cc-pVQZ/NormalPNO - DLPNO-CCSD(T)/cc-pVTZ/NormalPNO]
+ [DLPNO-CCSD(T)/cc-pVTZ/TightPNO - DLPNO-CCSD(T)/cc-pVTZ/NormalPNO]

The RMSE for DLPNO-CCSD(T)/cc-pVTZ/NormalPNO compared to this additive approximation is 0.5 kcal/mol.

Generation of geometries for the OSD4K:

For each heterocycle/nucleobase pair, we did the following. First, 54 initial structures were constructed by considering six orientations (generated by 60° rotations about the axis normal to the heterocycle) at nine equally spaced positions on a 2.8 x 2.8 Å grid in the plane 3.5 Å from the nucleobase. For non-symmetric heterocycles, 54 additional dimers were generated by flipping the heterocycle 180°. These 54 (or 108) initial structures were then optimized to the nearest energy minimum at the ωB97X-D/def2-TZVP level of theory[1, 2] under the constraint that the heavy atoms of each heterocycle remained in parallel planes. In total, we performed 23,760 constrained geometry optimizations, providing 3906 unique stacked dimers based on an RMSD cutoff of 0.4 Å.

MM Interaction Energies

Non-bonded interaction energies for GAFF, GAFF2, and Sage force fields[3-6] were computed for the dimers in the three datasets as follows. GAFF parameters were generated using AmberTools 22 as were the AM1-BCC, HF/6-31G(d), and wB97X-D/def2-TZVP RESP charges (paired with Gaussian 16 for the latter two). GAFF2 parameters and ABCG2 charges were generated using AmberTools24. Sage parameters were generated using OpenFF[7] and converted to the appropriate A-B form for the non-bonded potential given below. Interaction energies were then evaluated according to

$$E_{int}^{MM} = \sum_{i \in Nuc} \sum_{j \in Het} \left(C_R \frac{A_{ij}}{R_{ij}^{12}} - C_A \frac{B_{ij}}{R_{ij}^{6}} + \frac{q_i q_j}{R_{ij}} \right)$$

using the usual rules for combining the non-bonded parameters. The scaling parameters (C_R and C_A) are listed in Table 3 of the main text for each MM/charge model and were only applied to pairs of heavy atoms (*i.e.* $C_R = C_A = 1$ for any *ij* pair involving a hydrogen). Slightly better performance can be obtained by also scaling pairs involving hydrogens. However, the performance then degrades for heavily substituted heterocycles (data not provided). Similarly, further improvements, particularly with AM1-BCC charges, can be obtained by also scaling the Coulombic part of the interaction energy. However, this would preclude the application of this scaling procedure strictly to the aromatic component of a ligand because it would no longer preserve the total molecular charge.

Other Data

Cartesian coordinates for all structures in FRSD3K, NRSD4K, and OSD4K are provided in `ci4c02420_si_001.xyz`, `ci4c02420_si_002.xyz`, and `ci4c02420_si_003.xyz`, respectively.

All computed data can be found in `ci4c02420_si_004.xls`. The 'File' column in the spreadsheets is the name of each structure, which matches the comment lines in the XYZ files.

References

(1) Chai, J. D.; Head-Gordon, M. Long-range corrected hybrid density functionals with damped atom-atom dispersion corrections. *Phys. Chem. Chem. Phys.* **2008**, *10* (44), 6615-6620, 10.1039/B810189B. DOI: 10.1039/b810189b

(2) Weigend, F.; Ahlrichs, R. Balanced basis sets of split valence, triple zeta valence and quadruple zeta valence quality for H to Rn: Design and assessment of accuracy. *Phys. Chem. Chem. Phys.* **2005**, *7*, 3297-3305.

(3) Jakalian, A.; Jack, D. B.; Bayly, C. I. Fast, efficient generation of high-quality atomic charges. AM1-BCC model: II. Parameterization and validation. *J. Comput. Chem.* **2002**, *23* (16), 1623-1641. DOI: 10.1002/jcc.10128

(4) Boothroyd, S.; Behara, P. K.; Madin, O. C.; Hahn, D. F.; Jang, H.; Gapsys, V.; Wagner, J. R.; Horton, J. T.; Dotson, D. L.; Thompson, M. W.; et al. Development and Benchmarking of Open Force Field 2.0.0: The Sage Small Molecule Force Field. *J. Chem. Theory Comput.* **2023**, *19* (11), 3251-3275. DOI: 10.1021/acs.jctc.3c00039

(5) Wang, J.; Wolf, R. M.; Caldwell, J. W.; Kollman, P. A.; Case, D. A. Development and testing of a general amber force field. *J. Comput. Chem.* **2004**, *25* (9), 1157-1174. DOI: 10.1002/jcc.20035

(6) He, X.; Man, V. H.; Yang, W.; Lee, T. S.; Wang, J. A fast and high-quality charge model for the next generation general AMBER force field. *J Chem Phys* **2020**, *153* (11), 114502. DOI: 10.1063/5.0019056

(7) Eastman, P.; Swails, J.; Chodera, J. D.; McGibbon, R. T.; Zhao, Y.; Beauchamp, K. A.; Wang, L. P.; Simmonett, A. C.; Harrigan, M. P.; Stern, C. D.; et al. OpenMM 7: Rapid development of high performance algorithms for molecular dynamics. *PLoS Comput. Biol.* **2017**, *13* (7), e1005659. DOI: 10.1371/journal.pcbi.1005659
